# Shared Mechanisms for Mutually Exclusive Expression and Antigenic Variation by Protozoan Parasites

**DOI:** 10.3389/fcell.2022.852239

**Published:** 2022-03-08

**Authors:** Francesca Florini, Joseph E. Visone, Kirk W. Deitsch

**Affiliations:** Department of Microbiology and Immunology, Weill Cornell Medical College, New York, NY, United States

**Keywords:** mutually exclusive expression, antigenic variation, transcription, chromatin, nuclear organization

## Abstract

Cellular decision-making at the level of gene expression is a key process in the development and evolution of every organism. Variations in gene expression can lead to phenotypic diversity and the development of subpopulations with adaptive advantages. A prime example is the mutually exclusive activation of a single gene from within a multicopy gene family. In mammals, this ranges from the activation of one of the two immunoglobulin (Ig) alleles to the choice in olfactory sensory neurons of a single odorant receptor (OR) gene from a family of more than 1,000. Similarly, in parasites like *Trypanosoma brucei*, *Giardia lamblia* or *Plasmodium falciparum,* the process of antigenic variation required to escape recognition by the host immune system involves the monoallelic expression of *vsg*, *vsp* or *var* genes, respectively. Despite the importance of this process, understanding how this choice is made remains an enigma. The development of powerful techniques such as single cell RNA-seq and Hi-C has provided new insights into the mechanisms these different systems employ to achieve monoallelic gene expression. Studies utilizing these techniques have shown how the complex interplay between nuclear architecture, physical interactions between chromosomes and different chromatin states lead to single allele expression. Additionally, in several instances it has been observed that high-level expression of a single gene is preceded by a transient state where multiple genes are expressed at a low level. In this review, we will describe and compare the different strategies that organisms have evolved to choose one gene from within a large family and how parasites employ this strategy to ensure survival within their hosts.

## Introduction

Pathogenic organisms, including eukaryotic parasites, have evolved numerous mechanisms to ensure their survival in the different, often hostile environments they encounter as they transition through their complex life cycles. These diverse environments often include infection of multiple host species, each with different stresses that must be overcome for successful completion of the cycle. In particular, infected hosts often mount a vigorous immune response that can drastically reduce parasite numbers or eliminate the infection. One especially important mechanism for prolonged survival inside their host is the ability of parasites to respond to changing environmental conditions through alterations in gene expression. Several of the most dramatic responses involve the mutually exclusive expression of individual members of large, multicopy gene families. This process promotes clonal variability and enables populations of infecting parasites to rapidly adapt to the changing conditions that they encounter both while maintaining a chronic infection or while transitioning from one host to another. The ability to undergo clonal changes in gene expression is key for several processes that are vital for parasite survival, for example nutrient uptake, colonization of different tissues, host cell invasion and, perhaps most dramatically, immune evasion (Cortes and Deitsch, 2017). Mutually exclusive expression of genes encoding variable surface antigens is indeed the primary mechanism underlying the phenomenon of antigenic variation in parasites like *Trypanosoma brucei*, *Plasmodium falciparum*, and *Giardia lamblia* (Duraisingh and Horn, 2016). It enables them to periodically switch their antigenic signature and thereby escape recognition by the host immune system thus maintaining prolonged, chronic infections. Despite the relevance of this mechanism for parasite survival, little is understood regarding how this is achieved at a molecular level.

While mutually exclusive expression within large, multicopy gene families has been a high-profile subject of research within the parasitology community for many years, it is worth noting that this phenomenon is not a unique feature of pathogens, but rather a process conserved throughout the evolution of the eukaryotic lineage (Dalgaard and Vengrova, 2004; Goldmit and Bergman, 2004). Ranging from the simple choice between two alleles to the activation of a single gene within a larger family that can include thousands of copies, many of the basic mechanisms by which a single gene is chosen and expressed appear to be shared between even the most distant evolutionarily related organisms. For example, both *Trypanosoma brucei* and *Giardia lamblia* are referred to as early branching eukaryotes and are thought to be amongst the most evolutionarily divergent eukaryotes in existence today (Morrison et al., 2007; Lukes et al., 2014). Nonetheless, recent work suggests they share several molecular mechanisms for regulating multicopy gene expression with higher eukaryotes, including humans. In this review we describe how well-studied model organisms achieve mutually exclusive gene expression and explore analogies and differences with parasites.

## Examples of Mutually Exclusive Expression in Model Eukaryotes

Detailed molecular research into the mechanisms regulating mutually exclusive expression have often focused on higher eukaryotic organisms, with the yeast and mammalian model systems providing the majority of the conceptual insights. The genetic systems that are most relevant include *1*) the simple selection of one of two alleles for active transcription (mating type switching, immunoglobulin gene recombination and expression), *2*) the activation or silencing of entire chromosomes (dosage compensation) and *3*) single gene expression within large multicopy gene families (olfactory receptor gene expression). All these systems have proven to be rich sources of information that have influenced our understanding of similar gene expression systems in parasites.

### Yeast Mating-Type Switching

Both the fission yeast *Schizosaccharomyces pombe* and the budding yeast *Saccharomyces cerevisiae* are free-living, single-celled eukaryotes that can easily be grown in the lab and genetically manipulated. Under specific conditions, haploid cells of different mating types can fuse, resulting in diploid cells which can then undergo meiosis, sporulate and produce haploid cells again. While successful mating requires two cells of different mating types, individual cells can switch their mating type, between P and M cells for *S. pombe* or between a and α in *S. cerevisiae*, thus facilitating efficient creation of hybrids and exchange of genetic material. In these examples, the expressed mating type is determined by a transcriptional choice between two different alleles, thus representing a simple binary system of mutually exclusive expression.

**FIGURE 1:**
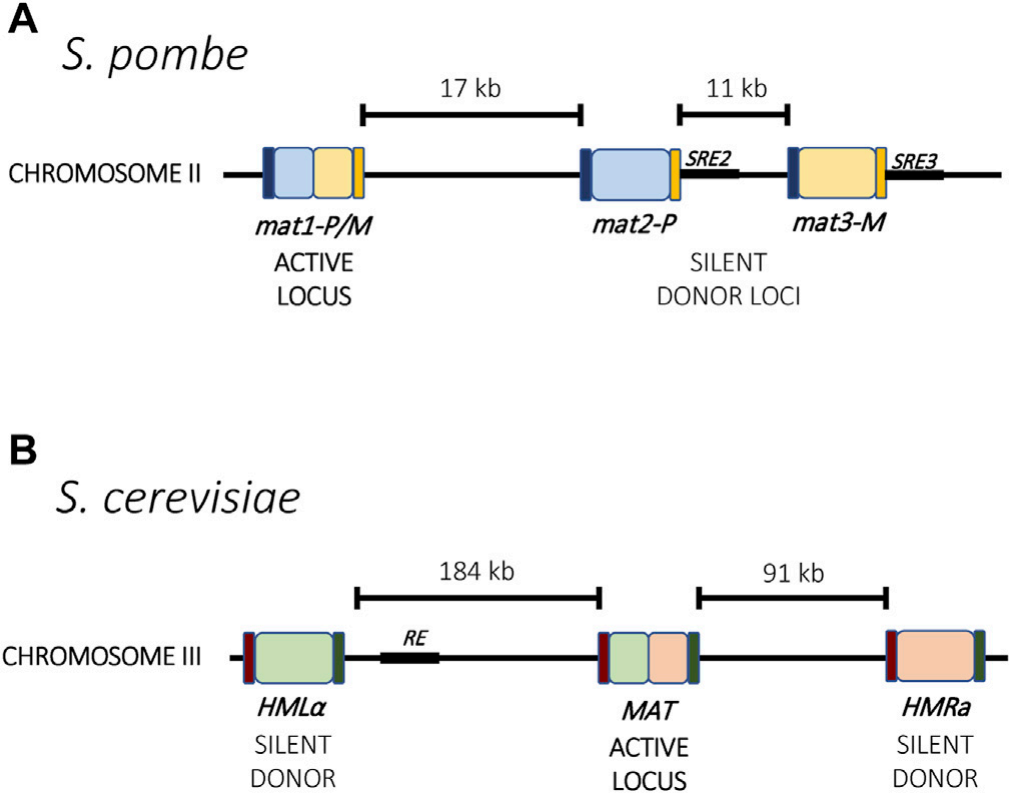
Mating type loci in yeast. **(A)** In *S. pombe*, the active locus *mat1* and two silent loci, *mat2-P* and *mat3-M*, reside on chromosome 2. Mating type switching results from recombination between one of the silent loci into the active locus. Each locus is flanked by the homology regions H1 (dark blue) and H2 (orange) that guide recombination. Two enhancer elements, SRE2 and SRE3 (black), interact with the Swi2-Swi5 complex and contribute to recombination choice. **(B)** In *S. cerevisiae*, the mating type loci are distributed across chromosome 3. Mating type switching results from recombination between the active *MAT* locus with either *HMLα* or *HMRa.* The Recombinational Enhancer (RE, black) directs recombination toward the HMLα locus. Homology regions for recombination are indicated in purple and green.

The mating-type switch is made possible through three gene-cassettes located on the same chromosome. In both *S. pombe* and *S. cerevisiae*, one cassette is constitutively transcriptionally active whereas the other two are silent and serve as donors for transposition into the active site (Figure 1). The mating type of the cell is determined by which of the silent cassettes occupies the transcriptionally active site, and mating type switching results from recombination using the alternative donor. Thus, through a recombinational mechanism, cells can switch between mating types. In *S. pombe*, the expressed cassette is referred to as *mat1* and can contain information copied from the two silent cassettes *mat2-P* and *mat3-M* (Figure 1A), while in *S. cerevisiae*, the content of the transcriptionally active MAT locus can be replaced with information from the silent loci *HMLα* and *HMRa* (Figure 1B) (Kelly et al., 1988; Haber, 1998). Additionally, the choice of which donor cassette is used for recombination is not random. For example, in *S. pombe*, the *mat2P* cassette gets chosen preferentially for recombination in M cells, whereas the *mat2-M* cassette is preferentially chosen in P cells, thereby increasing the overall probability of mating. A similar directionality is also observed in *S. cerevisiae* (Klar et al., 1982; Klar, 1990). These two single-celled organisms therefore provide an elegant model for mutually exclusive expression that couples transcriptional activation and silencing with genetic recombination.

### V(D)J Recombination of Immunoglobulin Genes

The mammalian immune system has evolved to recognize and destroy invading organisms through the continuous production of a vast repertoire of antigen receptors, called immunoglobulins, that are thought to be able to recognize virtually any possible antigen conformation. For this adaptive immune response to be effective it is necessary for the antigen receptor repertoire to be extremely diverse and that each individual receptor expressing cell only express a single immunoglobulin. Similar to mating type switching in yeast, this is achieved through a mechanism that incorporates DNA recombination and mutually exclusive expression of the genes encoding antigen receptors.

**FIGURE 2:**
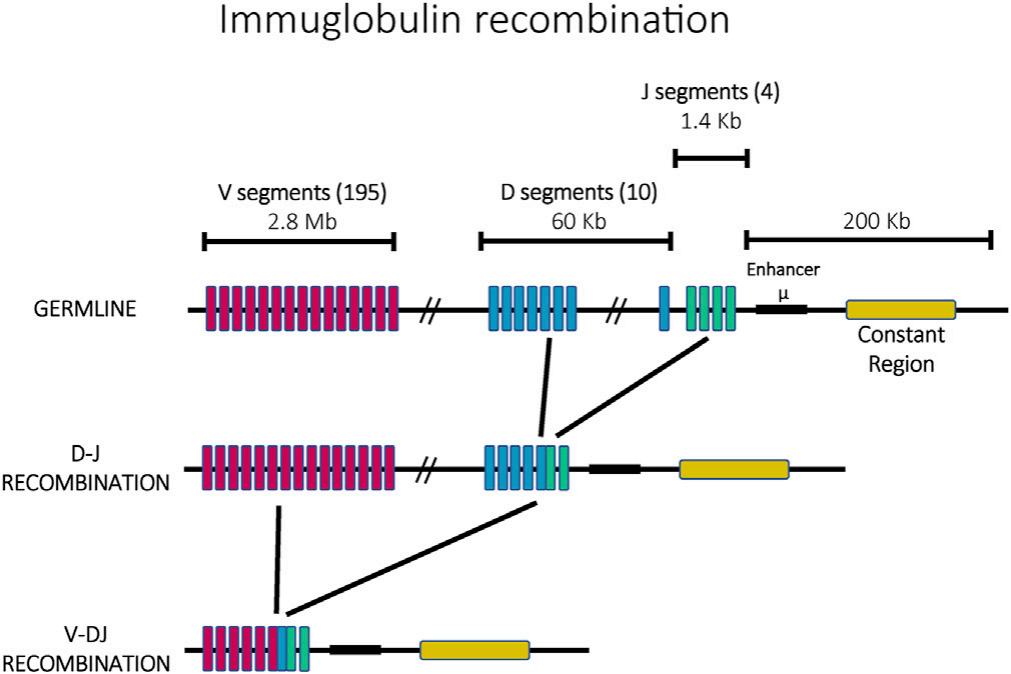
The ordered recombination of the mouse immunoglobulin heavy chain. The germline possesses 195 V segments (pink), 10 D segments (blue) and 4 J segments (green) in a locus of ∼3 Mb. In a first step, J and D segments recombine, followed by a second recombination with V segments.

Immunoglobulin diversification is generated through a mechanism called V(D)J recombination that ensures that every mature antigen receptor expressing cell expresses a different immunoglobulin. Each immunoglobulin is formed by two identical heavy chains and two identical light chains with each chain consisting of constant (C) and variable (V) regions. The process of creating a unique immunoglobulin begins from a germline containing array of highly similar gene fragments which can recombine to form single, functional open reading frames that encode a unique antigen receptor (Figure 2). The heavy chain is formed through the recombination of sets of Variable (V), Diversity (D) and Joining (J) genes, whereas the light chain is formed by rearranged V and J genes (Weigert et al., 1978; Early et al., 1980). In mice, the immunoglobulin locus contains 195 V segments, 10 D segments and 4 J segments arranged in tandem within a chromosomal region ∼3 MB long. Epigenetic mechanisms control the order and the site of recombination. Specifically, recombination begins with one heavy chain allele, chosen randomly. If the recombination events result in the creation of a functional heavy chain, only this allele is actively transcribed and the second allele is permanently silenced. In contrast, if the recombination events do not yield a functional heavy chain, the second allele is accessed and recombined in an attempt to generate a functional protein. Only when a functional heavy chain has been generated does a similar recombination occur at the light chain alleles, with a similar feedback mechanism ensuring that only a recombined allele that encodes a functional receptor gets expressed in mature immune cells (*see* (Jung and Alt, 2004) for a review of this process). Similar to mating type loci switching in yeast, mutually exclusive expression (also called allelic exclusion in this system) is linked to DNA recombination, however here the recombination events have the additional function of diversifying the sequence of the resulting protein, thus serving as a continuous source of variability in antigen recognition and maintaining the enormous breadth of the repertoire of antigen binding receptors.

### Inactivation of X-Chromosome

X-inactivation is an example of mutually exclusive gene expression on a chromosome-wide scale. The sex of mammals is determined by the XX/XY sex-determination system, with differentiation into the male sex determined by genes present on the Y chromosome (Graves, 1995; Lahn and Page, 1997). In females, the presence of two X chromosomes would result in a potentially lethal dose of expression of X-linked genes if the alleles present on both chromosomes were equally transcribed. To ensure proper dosage compensation, one of the X chromosomes is transcriptionally silenced (Lyon, 1961). This silenced X chromosome is condensed into heterochromatin and forms a compact structure within the nucleus called a Barr body (Barr and Bertram, 1949; Boumil and Lee, 2001). This inactivation can be either imprinted or random, depending on the species. Imprinted X-inactivation preferentially silences the paternal X chromosome while in random X-inactivation systems there is an equal chance of paternal or maternal X-inactivation, a cell fate choice that occurs early during embryonic development. Once inactivation has occurred, all resulting cells throughout the lineage will maintain this transcriptional state, resulting in a mosaic pattern of expression in the resulting organism (Graves, 2006; Augui et al., 2011).

**FIGURE 3:**
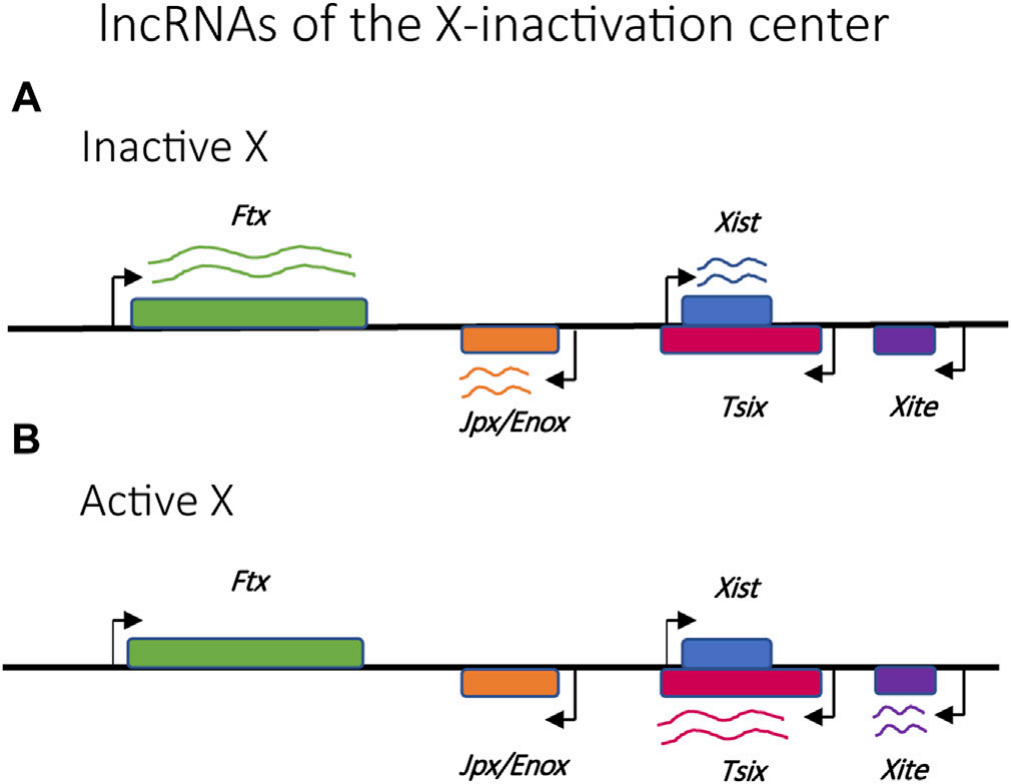
Relative positions of selected lncRNAs implicated in the regulation of X-chromosome activation and silencing. **(A)** *Ftx*, *Jpx/Enox* and *Xist* lncRNAs are upregulated in the inactive X chromosome. **(B)** *Tsix* and *Xite* lncRNAs are upregulated in the active X chromosome.

Random X-inactivation is initiated by competition between transcriptional promoters within a specific locus on each X chromosome called the X-inactivation center (Xic) (Figure 3) (Lee et al., 1996). This locus is responsible for the production of several long noncoding RNAs (lncRNAs), the most prominent of which are Xist and Tsix (Lee et al., 1999). Stable expression of Xist only occurs on the chromosome destined to be silenced, where it is incorporated into the structure of the chromatin along the full length of the chromosome (Plath et al., 2002; Engreitz et al., 2013). The presence of the Xist RNA is key to initiating the assembly of transcriptionally silent heterochromatin and the partitioning of the silent X-chromosome into the Barr body (Avner and Heard, 2001; Augui et al., 2011). The role of the Xist transcripts represents one of the first examples of how the production of lncRNAs is often the initiating event for establishing mutually exclusive expression.

### Olfactory Receptor Gene Expression

The sense of smell, specifically the ability to ascertain environmental odorants, is facilitated by olfactory receptors (OR). In vertebrates, odorants are detected by a collection of G protein-coupled receptors displayed along the cilia and synapses of the olfactory sensory neurons (OSNs) making up the OR family (Buck and Axel, 1991; Monahan and Lomvardas, 2015). There is a wide range of variability in the number of different ORs encoded within the genomes of various vertebrates, with humans possessing ∼400 OR genes and mice ∼1,000 (Buck and Axel, 1991; Niimura and Nei, 2007). Mutually exclusive expression, with each OSN expressing a signal OR, is essential to the functionality of the olfactory system (Chess et al., 1994). Each OR is capable of binding a wide variety of odorants at different affinities resulting in a specific odorant’s detection by a unique combination of ORs. The aggregate of the resulting signals from the excited neurons is then processed in the olfactory bulb and cortex, thereby providing crucial sensing of the odorants present in the surrounding environment (Malnic et al., 1999; Monahan and Lomvardas, 2015).

**FIGURE 4:**
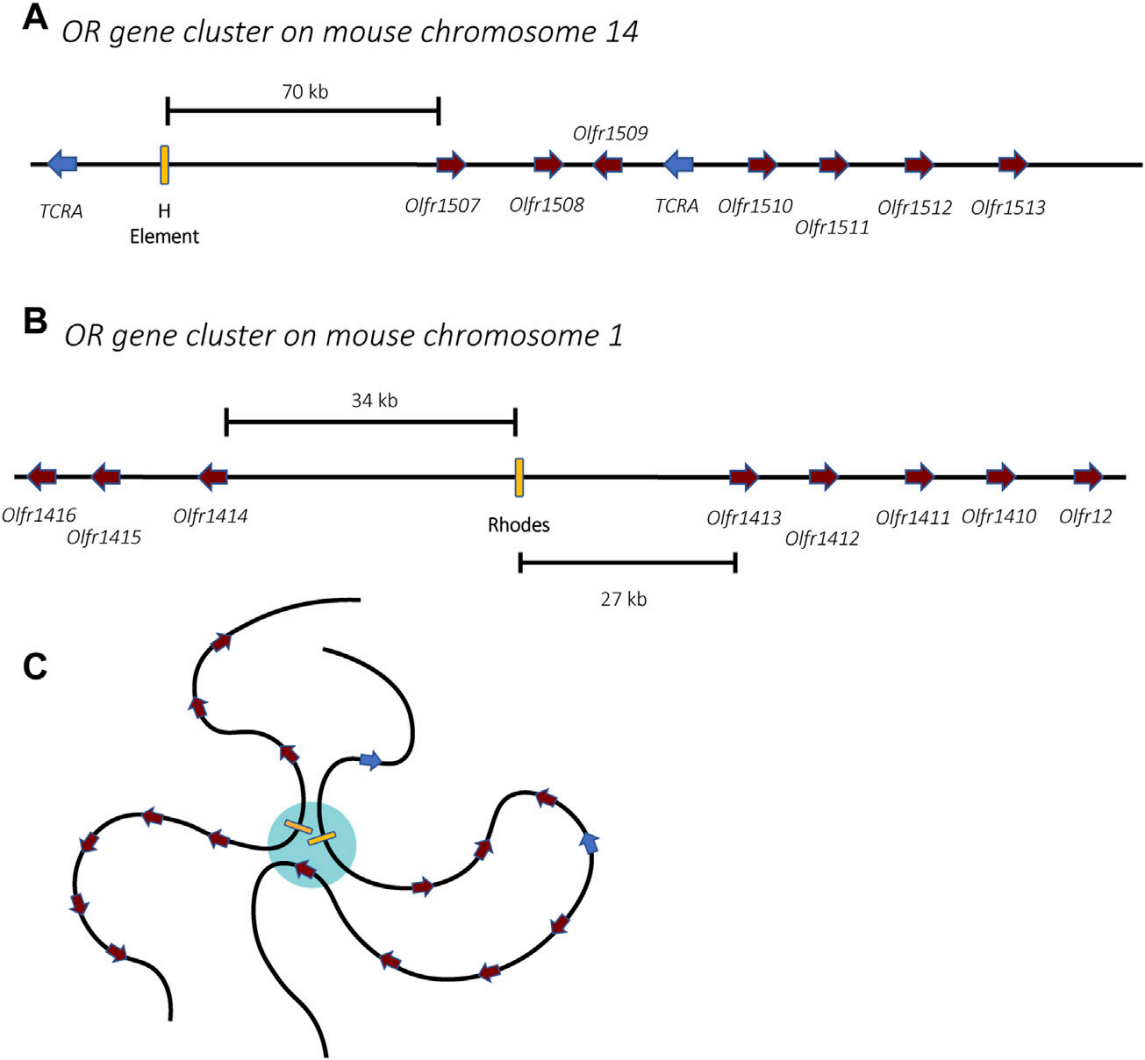
Organization of the olfactory receptor gene clusters in mice. Two gene clusters are shown, one from chromosomes 14 **(A)** and one from chromosome 1 **(B)**. The olfactory receptor genes are shown in burgundy while unrelated genes encoding T-cell receptor alpha chains are shown in blue. Enhancer elements, also called Greek Islands, are shown as yellow boxes. **(C)** In mature olfactory neurons, the enhancer elements associate within a phase-separated region of the nucleus (blue) that enables active transcription of a single gene. For simplicity, association of the two enhancers from **(A**,**B)** is shown, however in mature olfactory neurons, enhancers from all 63 receptor gene clusters might associate, forming a single super-enhancer hub.

Members of the OR gene family are found throughout the genome, organized into clusters of genes on most chromosomes (Figures 4A,B) (Glusman et al., 2001; Niimura and Nei, 2003). During OSN differentiation and maturation, the chromosomes undergo a systematic nuclear reorganization that results in the physical interaction of the OR gene clusters into phase-separated regions of the nucleus (Figure 4C). This organization is anchored by specific genetic elements at each cluster of OR genes called “Greek Islands.” These elements appear to bring the OR genes together and thus play a pivotal role in the nuclear reorganization that enables mutually exclusive expression within this gene family (Lomvardas et al., 2006; Bashkirova and Lomvardas, 2019; Monahan et al., 2019; Pourmorady and Lomvardas, 2021). This nuclear reorganization occurs in a stepwise fashion during OSN differentiation, initially leading to low-level expression of many OR genes prior to high-level, exclusive expression of a single gene in fully differentiated cells (Hanchate et al., 2015; Tan et al., 2015). These discoveries have proven to be important concepts that likely apply to many other multicopy gene families in distantly related organisms, including parasites.

## Mutually Exclusive Expression in Parasites: Antigenic Variation

**FIGURE 5:**
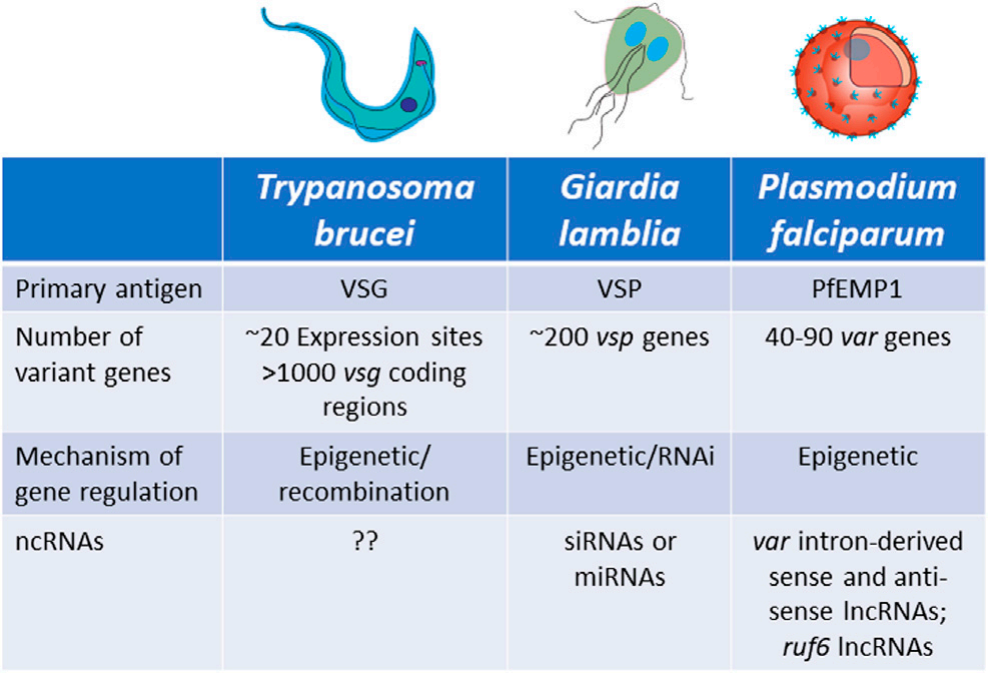
Characteristics of antigenic variation by mutually exclusive gene expression in *Trypanosoma brucei*, *Giardia lamblia* and *Plasmodium falciparum*.

To maintain an infection within a mammalian host, pathogens must be able to evade the immune response, including the production of highly specific antibodies that recognize surface antigens unique to the pathogen. Despite vast evolutionary distances, many eukaryotic parasites have evolved very similar strategies, specifically the development of large, multicopy gene families in which each gene encodes a protein of similar function but that is antigenically distinct. By expressing a single member at a time and systematically cycling through the family over the course of an infection, parasites can perpetuate chronic infections of remarkable length. Mechanisms that establish and maintain mutually exclusive expression are imperative for the success of this type of immune avoidance mechanism. The gene regulatory processes underlying antigenic variation are also used to regulate other biological processes that require clonally variant gene expression, most notably alternative invasion pathways or altered nutrient uptake in *Plasmodium*. However here we will focus on the large gene families involved in antigenic variation in African trypanosomiasis, human malaria and giardiasis (Figure 5).

### Trypanosoma brucei

**FIGURE 6:**
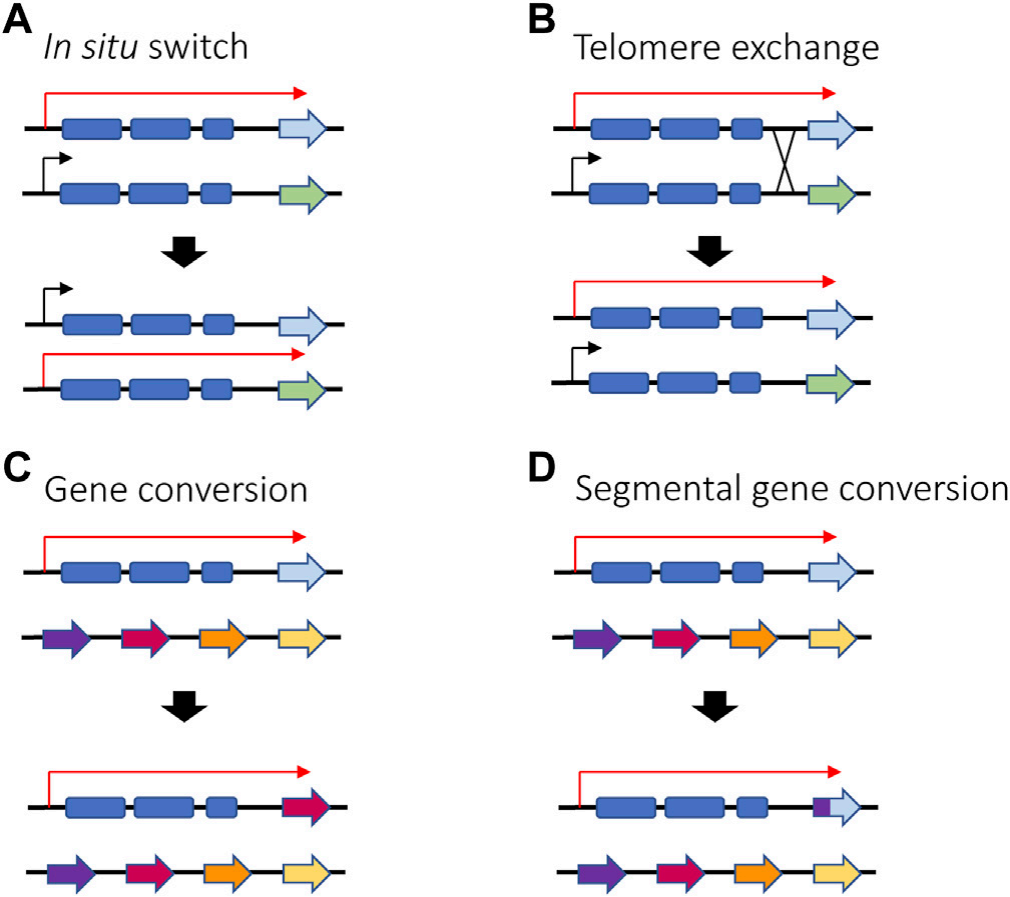
Different ways in which VSG switching can be achieved in *T. brucei in situ* switching **(A)** where one promoter is silenced and another one is activated relies on epigenetic changes and is the only mechanism that does not involve recombination. Telomere exchange **(B)** or gene conversion **(C**,**D)** require DNA recombination and gene rearrangement, either at the telomeres level or within the polycistronic unit. If gene conversion occurs, the active gene is lost, and a new gene is copied into the active ES. Red arrows represent active ES promoters while black arrows represent silent ES promoters.

*Trypanosoma brucei* is a unicellular parasite responsible for sleeping sickness in humans and nagana in cattle. Its life cycle alternates between tsetse flies and mammalian hosts, where they live extracellularly in the bloodstream and other tissues. When infecting mammals, the parasite’s surface coat consists of a single antigen called the variant surface glycoprotein (VSG), which forms a thick layer that effectively obscures other surface molecules from recognition by the host immune system. Key to long-term infection is antigenic variation of this coat: to escape clearance by host antibodies, bloodstream form trypanosomes periodically switch the VSG that they express. This is possible thanks to an abundant genomic repertoire accounting for more than 1,000 *vsg* genes or gene fragments (Horn, 2014). The mRNA encoding the active VSG is transcribed from a specific subtelomeric locus called an Expression Site (ES). The *T. brucei* genome contains around 20 ES, but only one is expressed at a time, ensuring that only one VSG is displayed on the surface of every parasite. Thus, mutually exclusive expression in this organism refers to expression of a single *vsg* ES (Cross, 1975; Kooter et al., 1987). VSG switching can either be *in situ*, where one ES promoter is silenced while another is activated, or by a recombination event that copies a silent gene (or portion of a silent gene) into the active ES (Li, 2015) (Figures 6A–D).

African trypanosomes are evolutionarily very distant from the higher eukaryotes in which most molecular mechanisms controlling transcription were initially defined and consequently display many unusual characteristics. For example, unlike standard models of eukaryotic transcription in which each protein coding region is contained within an individual gene, each ES is a polycistronic unit of 45–60 kb containing multiple ES-associated genes (ESAGs) and a promoter typically located ∼50 kb upstream of the *vsg* coding region (Glover et al., 2013). Interestingly, polycistronic organization of genes is not unique to the ES, but rather is a genome-wide feature that characterizes this ancient lineage of parasites (Clayton, 2019). Another peculiarity of *vsg* transcription is that it is carried out by RNA Polymerase I, a unique example of an RNA Pol I transcribed mRNA among eukaryotes (Gunzl et al., 2003). Nonetheless, the basic strategy of employing mutually exclusive expression within a large gene family is similar to what is observed in higher eukaryotes.

Interestingly, *vsg* transcription starts before parasites enter the mammalian host in the last phase of their development within the tsetse fly, called the metacyclic stage. At this point of their lifecycle, trypanosomes express the metacyclic form of the variant surface glycoprotein (mVSG), as a type of pre-adaptation to their entry into the mammalian host. Similar to mutually exclusive expression of a single *vsg* ES, only one of approximately eight mVSG encoding genes is transcribed in each metacyclic parasite, thereby ensuring heterogeneity in the population and increasing the probability of a successful infection (Barry et al., 1998; Ramey-Butler et al., 2015; Muller et al., 2018). While similarly displaying mutually exclusive expression, unlike the *vsg* ESs of the bloodstream form of the parasite, the mVSG genes are the only example in trypanosomes of monocistronic transcription of a protein coding gene (Ginger et al., 2002).

### Plasmodium falciparum

*Plasmodium falciparum* is the parasite responsible for the vast majority of cases of malaria around the world and it is transmitted between people by *Anopheles* mosquitoes. The *P. falciparum* genome encodes several families of clonally variant genes involved in numerous processes including antigenic variation, erythrocyte invasion and erythrocyte permeability (Cortes and Deitsch, 2017). Other malaria species, including the model parasites that infect rodents, also have clonally variant gene families that display variable expression (Otto et al., 2014; Brugat et al., 2017; Lin et al., 2018). However, the lion’s share of research into transcriptional regulation and mutually exclusive expression has investigated the *var* gene family. Therefore, for the purpose of this review we will focus our attention on *var* genes. Upon entry into the human host, after initial replication inside hepatocytes the parasites are released into the bloodstream where they invade and replicate within erythrocytes (Cowman et al., 2016). Once inside the erythrocytes, the parasites make extensive modifications to the host cell, including alterations to the cytoskeleton and insertion into the erythrocyte membrane of a protein called *Plasmodium falciparum* Erythrocyte Membrane Protein 1 (PfEMP1) (Boddey and Cowman, 2013). This protein is exposed on the erythrocyte surface where it binds to ligands on the vascular endothelium, enabling the infected cells to cytoadhere within capillaries and sequester away from the peripheral circulation. This prevents the infected cells from being cleared by the spleen. However, by exposing PfEMP1 on the erythrocyte surface, the parasite is now vulnerable to the antibody response of its host. To escape recognition, *P. falciparum* parasites systematically change the expressed PfEMP1, thereby undergoing antigenic variation in a way analogous to *T. brucei* and *G. lamblia*. PfEMP1 is encoded by a multicopy family of genes called *var* (Scherf et al., 1998; Deitsch and Dzikowski, 2017). Unlike *T. brucei*, the repertoire of *var* genes is relatively small, limited 40–90 genes per genome, depending on the isolate (Otto et al., 2018b). Similar to *vsg* expression, *var* gene expression is mutually exclusive and regulated at the level of transcription initiation.

**FIGURE 7:**
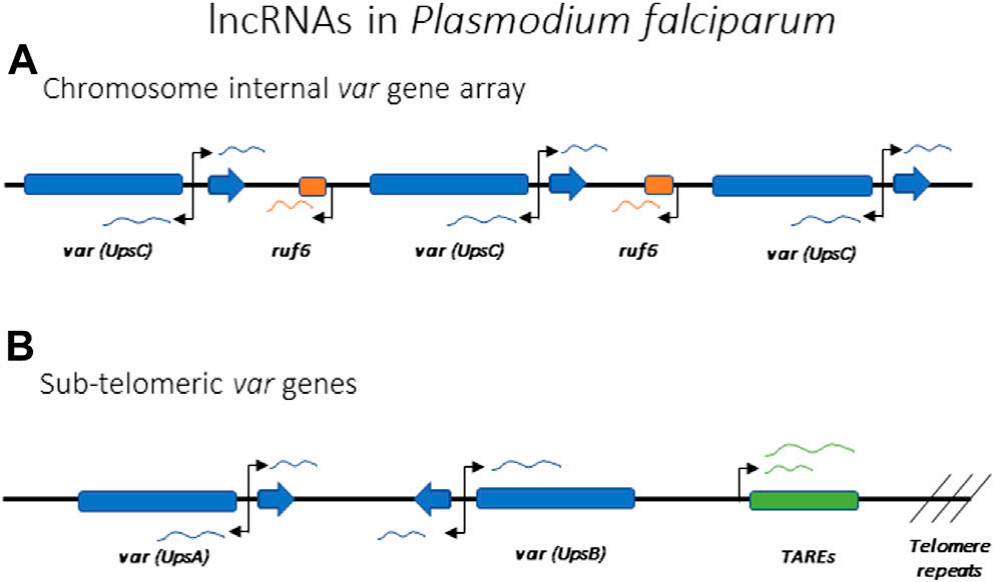
lncRNAs implicated in the regulation of antigenic variation in *Plasmodium falciparum.* Promoters driving the lncRNAs are indicated by arrows. **(A)** Schematic of a representative *var* gene array found within an internal region of a chromosome. Individual *var* genes can each express intron derived lncRNAs in both the sense and antisense directions. Chromosome internal *var* genes are often separated by *ruf6* elements that also express lncRNA. **(B)** Schematic representation of subtelomeric *var* genes with *var* intron derived ncRNA and lncRNA derived from TARE elements indicated.

All *var* genes have a common bi-exonic structure, with the first exon encoding the extracellular portion of PfEMP1 and the second coding for the cytoplasmic portion, with a similar sequence among all *var* genes (Figure 7). Each gene possesses two promoters: one located approximately 1 kb upstream of the coding region and subject to mutually exclusive activation, and a second within the intronic region. The second promoter is bi-directional and drives the expression of sense and anti-sense lncRNAs (Figure 7) (Calderwood et al., 2003; Epp et al., 2009). The majority of the *var* gene family is subject to frequent recombination, resulting in the gene family displaying tremendous sequence diversity when the repertoire of *var* genes from different isolates are compared. However, two genes, referred to as *var1csa* and *var2csa*, appear to be conserved in all *P. falciparum* isolates from around the world and are also found in the related Plasmodium species that infect chimpanzees and gorillas (Otto et al., 2018b; Gross et al., 2021). It has been suggested that these genes could serve an additional function as conserved regulatory elements for coordinating mutually exclusive expression (Mok et al., 2008; Ukaegbu et al., 2015).

### Giardia lamblia

**FIGURE 8:**
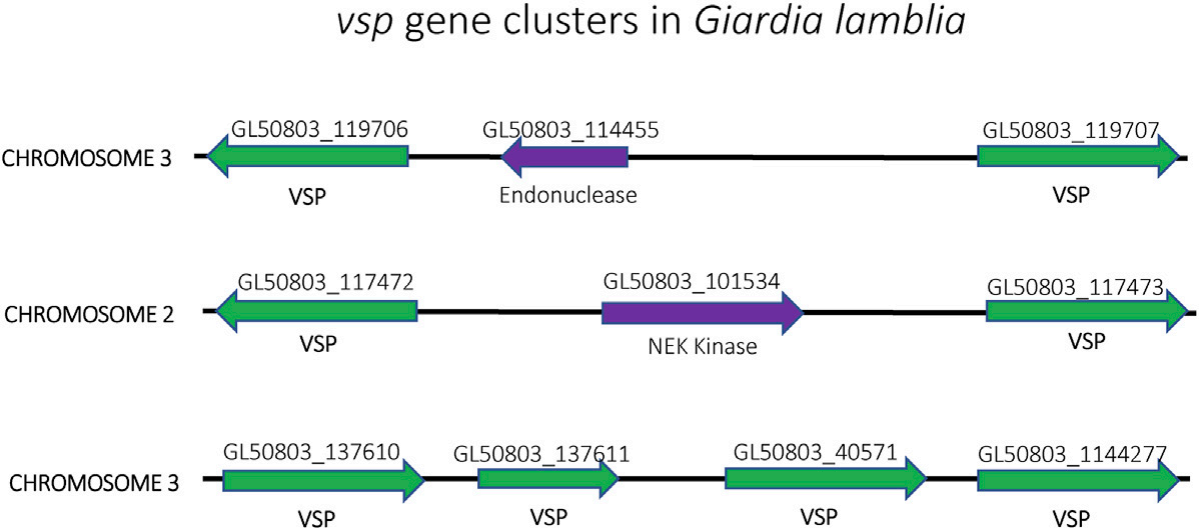
Organization of the *vsp* gene family in *Giardia lamblia*. *vsp* genes (green) from chromosomes 2, 3 and 4 are shown. The genes are often found in head-to-head orientation separated by an unrelated gene (purple) (top and middle) or in a linear arrangement of head-to-tail oriented genes (bottom). Gene annotation numbers from GiardiaDB.org are shown above each gene.

Similar to *T. brucei*, *Giardia lamblia* is an early-branching eukaryotic parasite that is evolutionarily very distant from higher eukaryotes. It infects the intestines of its vertebrate hosts and is one of the major causes of intestinal diseases and diarrhoea throughout the world. It is binucleated, with each diploid nucleus possessing a compact genome of around 12 Mb. *Giardia*’s life cycle alternates between two forms: a motile trophozoite which colonizes the upper intestine and an infective cyst form that enables infection of new hosts through oral-faecal transmission (Adam, 2001). The trophozoite form is coated with a variant-specific protein, VSP, which, like VSG in *T. brucei*, serves as the dominant antigen recognized by the host immune system, resulting in a strong antibody response. To avoid antibody mediated clearance, these parasites can switch the expressed form VSP through mutually exclusive expression from a repertoire of around 200 *vsp* genes arranged as individual genes or in tandem arrays throughout the parasite’s genome (Figure 8). This enables them to display antigenic variation in a way similar to *P. falciparum* or *T. brucei* (Nash and Aggarwal, 1986; Nash, 2002). However, the binucleated nature of *Giardia* poses unique problems for mutually exclusive expression. Since both nuclei are transcriptionally active and functional, it is important that *vsp* expression is coordinated between the two nuclei so that only a single VSP is ultimately expressed on the surface of the parasite. Giardia appears to accomplish this feat by using an RNAi-like mechanism within the cytoplasm to degrade nearly all *vsp* transcripts from both nuclei. Only mRNA from a single *vsp* gene escapes degradation, thus leading to expression of a single VSP on the parasite’s surface. What enables transcripts from a single *vsp* gene to avoid destruction is not understood, although it appears to depend on orthologues of the RNAi machinery (Prucca and Lujan, 2009; Gargantini et al., 2016). Thus, in this system, mutually exclusive expression is rooted in mRNA stability rather than transcriptional activation and silencing, although the ultimate result of antigenic variation is the same.

## Shared Mechanisms for Mutually Exclusive Expression

Ranging from the model yeasts and mammalian systems to the protozoan parasites, these organisms represent an exceptionally broad portion of the eukaryotic evolutionary tree. Nonetheless, many of the molecular mechanisms that underpin mutually exclusive expression are shared, suggesting that they are rooted in the origins of the eukaryotic lineage. By using a comparative approach, it is possible to gain insights into the molecular components and key players that regulate these important processes and to see how each organism employs these tools to solve specific evolutionary problems.

### Master Genetic Elements

One characteristic shared by all examples of mutually exclusive gene expression is the presence of non-coding master genetic elements that influence chromatin assembly and transcription at the loci. These include DNA sequence elements, transcriptional enhancers and non-coding RNAs (explored in more detail in section 4.2). For example, in *S. pombe*, two enhancers are responsible for directionality of the donor choice during mating type switching. The enhancers SRE2 and SRE3 are located next to the two silent donors, *mat2-P* and *mat3-M*, respectively, and removal of one skews the choice in the direction of the opposite donor (Figure 1A). These enhancers guide the interaction of the Swi2-Swi5 complex with the local chromatin, which in turn can recruit Rad51 and guide recombination of the locus (Jia et al., 2004; Jakociunas et al., 2013). A similar phenomenon has been described for *S. cerevisiae* involving an element named the Recombination Enhancer (RE). The RE is located next to the HML locus and directs recombination towards that locus (Figure 1B). When deleted, selection of the HML locus for recombination is dramatically reduced (Wu and Haber, 1996). Unlike *S. pombe*, the two donors in *S. cerevisiae* are located at the two opposite ends of the chromosome, so enhancer activation and silencing likely also involve chromatin rearrangement to bring the loci together for recombination.

Similarly, the choice of which olfactory receptor gene is expressed involves specific enhancer elements located adjacent to clusters of olfactory receptor genes. The first of these to be identified, termed the H element (Figure 4A), is a 2-kb homology region conserved between mouse and human sequences that was found to be essential for *cis* activation of genes on transgenic yeast artificial chromosomes (Serizawa et al., 2003). Using Chromosome Conformation Capture (3C) and DNA/RNA fluorescence *in situ* hybridization (FISH), the H element was found to interact with OR gene promoters from several different chromosomes and to colocalize specifically with the active OR allele, suggesting it could serve as a singular *trans* acting element essential for monoallelic expression (Lomvardas et al., 2006). However, deletion of the H element only affected expression of the OR genes found in the adjacent cluster, and there was no global effect on olfactory receptor expression (Fuss et al., 2007). This prompted a search for additional enhancer elements, which led to the discovery of a total of 63 OR enhancers (also called “Greek Islands”), each found adjacent to OR gene clusters (Figure 4B) (Markenscoff-Papadimitriou et al., 2014; Pourmorady and Lomvardas, 2021). These enhancers are proposed to lead to the formation of a subnuclear olfactory receptor compartment through the actions of the chromatin binding proteins Lhx2, LDB1 and EBF, where they form a single super-enhancer hub that associates specifically with the single active olfactory gene (Figure 4C) (Monahan et al., 2019). While it has been demonstrated that the formation of this enhancer hub is essential for OR gene transcription, its role in the selection of the active OR remains unclear.

It has been shown that in *T. brucei* and *P. falciparum* the genes involved in antigenic variation also cluster together in specific subnuclear locations, however no specific enhancers or genomic elements have yet been identified. Nevertheless, recent advances in genome-wide analysis are beginning to provide insights into potential elements that could play a role in this process. In *T. brucei*, the unique role of RNA Pol I in transcribing the active *vsg* gene sets it apart from other mRNA encoding genes in the parasite’s genome. However, it has been demonstrated that the *vsg* promoter sequence can be replaced with a rRNA promoter and still be properly regulated and transcribed in a mutually exclusion fashion (Rudenko et al., 1995). This suggests that the mechanisms of recognition for transcriptional control are more dependent on chromosomal context and positioning near the telomere than on the promoter sequence itself. Recent Hi-C analysis confirmed that the silent ESs cluster within the nucleus, while the active ES localizes to a unique subnuclear structure called the expression site body (Muller et al., 2018). The active *vsg* gene also interacts with a specific locus on chromosome nine where the spliced-leader (SL) RNA array is located (Faria et al., 2021). This locus consists of a tandem array of ∼200 SL RNA genes, each with its own promoter, encoding the SL-RNA that is *trans*-spliced to 5′ end of each trypanosome mRNA. This stabilizes the transcripts and provides them with a cap structure (Nelson et al., 1983; Perry et al., 1987). It is proposed that the close proximity of the active ES with the SL-RNA array acts as a post-transcriptional enhancer, ensuring high turnover of SL RNAs to more efficiently produce mature *vsg* mRNAs.

Using both FISH (Figueiredo et al., 2002; Duraisingh et al., 2005) and Hi-C (Ay et al., 2014), *var* genes in *P. falciparum* have been shown to cluster in specific nuclear compartments, with the active *var* gene being separated from the silent loci, but no specific genetic elements have been connected to this clustering. As previously mentioned, there are two *var* genes, *var1csa* and *var2csa*, that are uniquely conserved amongst all isolates of *Plasmodium falciparum*, and this conservation extends to related species that infect chimpanzees and gorillas (Gross et al., 2021). This is in stark contrast to the highly polymorphic sequences of all other *var* genes, leading to speculation that these two genes could play a role in organization of the family or by directly regulating transcription (Mok et al., 2008; Ukaegbu et al., 2015). Two other genetic elements that seem to be important for *var* gene activation and silencing are the promoter regions upstream of each gene and the conserved intron that interrupts all *var* coding regions. *var* promoters can be classified into five groups (UpsA, B, C, D and E), with the UpsA and B types found within subtelomeric regions of the chromosome while the UpsC promoters are found at genes located in the interiors of the chromosomes (Figures 7A,B). UpsD and E are specific to *var1csa* and *var2csa*, respectively. While the different promoter types display somewhat different rates of transcriptional activation and silencing, all appear to be co-regulated in terms of mutually exclusive expression. Experiments employing episomal constructs have shown that *var* promoters must be paired with a *var* intron to be subject to mutually exclusive expression (Deitsch et al., 2001; Frank et al., 2006; Dzikowski et al., 2007), and this interaction is dependent on specific pairing elements (PEs) located within the upstream region of every *var* gene and the intron (Avraham et al., 2012). The precise function of these interactions is unknown, although the intron has been shown to be the source of non-coding RNAs implicated in regulating *var* gene transcription.

### Non-Coding RNAs

An additional layer of control common to several organisms that employ mutually exclusive expression is the involvement of non-coding RNAs. One of the first and best studied examples of lncRNAs involved in mutually exclusive expression is the X-inactive-specific transcript (*Xist),* which is required for X chromosome inactivation (XCI) (Penny et al., 1996; Wutz and Jaenisch, 2000). The minimal chromosomal region required to ensure XCI is referred to as the x-inactivation centre (Xic) (Augui et al., 2011) which contains the *Xist* sequence as well as several other key lncRNAs that act as regulators of *Xist*, including *Tsix*, *Ftx*, *Jpx/Enox* and *Xite* (Figure 3) (Ogawa and Lee, 2003; Froberg et al., 2013; Maclary et al., 2013; Loda and Heard, 2019)*.* During embryonic development, the *Xist* transcript is incorporated into the chromatin structure of the inactive X chromosome, spreading from the Xic and “painting” the entire chromosome. This initiates the assembly of condensed heterochromatin and the segregation of the inactive X into the Barr body. In the mouse model, *Tsix* is transcribed from the Xic in the antisense direction as *Xist* and serves to prevent incorporation of *Xist* into the chromatin structure of the active X chromosome. Thus, this interplay of lncRNAs is key to the choice of which X chromosome becomes silenced and for the assembly of the condensed chromatin required to suppress gene expression.

Non-coding RNAs have also been implicated in the regulation of V(D)J recombination at the immunoglobulin loci. The immunoglobulin locus was one of the first examples of ncRNAs, at the time described as “sterile” transcripts. Strikingly, these transcripts are produced only from the chromosomal regions that are poised for recombination (Lennon and Perry, 1985). It has been proposed that transcription of these ncRNAs leads to opening of the chromatin structure and accessibility for recombination (Corcoran, 2010). Indeed, transcription of lncRNAs precedes each recombination event, with lncRNA transcription and recombination occurring first at the site of DJ recombination, then subsequently from the V segments. Both sense and antisense transcripts are produced, with transcripts coming from the coding region of V segments and antisense transcripts from the intergenic regions.

While the exact mechanisms are unclear, non-coding RNAs are thought to also play important roles in *P. falciparum* and *G. lamblia* in regulation of antigenic variation. Both sense and antisense lncRNAs are known to be produced from a bi-directional promoter within the intron of each *var* gene in *P. falciparum* (Figure 7) (Epp et al., 2009). The antisense lncRNA is 1.7 kb long and it is only expressed from the active *var* gene, early in the replicative cycle at the same time when the *var* mRNA is expressed. Interestingly, episomal expression of the antisense transcript can activate a silent *var* gene (Amit-Avraham et al., 2015), suggesting this lncRNA is involved in activating the locus. The sense lncRNA is 2.5 kb long and it is expressed from the exon 2 of all *var* genes late in the cycle when *var* genes are normally all silenced (Calderwood et al., 2003; Kyes et al., 2003). No clear function has yet been demonstrated for this lncRNA, but it remains nuclear and is associated with chromatin, suggesting it could function in *var* gene silencing. Another class of lncRNAs proposed to play a role in *var* gene regulation are named RUF6 (RNA of Unknown Function-6). These are transcribed from 15 genes that are dispersed among the chromosome-internal *var* gene arrays (Figure 7A). They have a conserved sequence with an unusual GC-rich content compared to the rest of *P. falciparum* genome, which has <20% GC-content (Gardner et al., 2002; Upadhyay et al., 2005). Although their mechanism of action is unknown, FISH showed they localize close to *var* genes and their expression has been linked to activation of specific *var* genes (Wei et al., 2015; Guizetti et al., 2016; Barcons-Simon et al., 2020). Within the subtelomeric regions, UpsA and B *var* genes are separated from the chromosome end by Telomere Associated Repeat Elements (TARES) that are transcribed into lncRNAs (Broadbent et al., 2011; Sierra-Miranda et al., 2012) (Figure 7B). The function of these lncRNAs is not known, although they have been proposed to contribute to *var* gene regulation. Thus, similar to X-inactivation, there appear to be multiple lncRNAs involved in regulating *var* gene activation, silencing and mutually exclusive expression. The study of lncRNAs in model organisms indicates that they often bind chromatin and drive gene expression and chromatin rearrangement (Li and Fu, 2019), therefore a role for lncRNAs in the regulation of the *var* family seems likely.

Non-coding RNAs have also been proposed to play a key role in regulating antigenic variation in *G. lamblia*. Due to the presence of two nuclei and thus the need to coordinate the choice of which *vsp* gene is expressed to attain mutually exclusive expression, *Giardia* evolved a post-transcriptional control mechanism rather than regulating activation and repression at the level of transcription. Two different classes of small RNAs have been implicated in *vsp* control: small interfering RNAs (siRNAs) or micro-RNAs (miRNAs) (Prucca et al., 2008; Saraiya et al., 2014). Elimination of components of the RNA interference (RNAi) machinery, such as Dicer or RNA-dependent RNA Polymerase (RdRP), disrupts mutually exclusive *vsp* expression and results in trophozoites expressing more than one VSP on their surface. These experiments are consistent with a model in which all *vsp* genes are actively transcribed, but that a cytoplasmic complex including Argonaute, Dicer and the RdRPN limits expression to a single *vsp* mRNA, through the production of either miRNAs or siRNAs (Prucca et al., 2008; Saraiya et al., 2014). In either case, it has been proposed that the *vsp* mRNA that is ultimately stabilized and actively translated is determined by a threshold in the amount of transcript available in the cytoplasm. There is also evidence for a role for epigenetic mechanisms controlling the level of transcription from each *vsp* gene, thus contributing to which transcript reaches the threshold (Kulakova et al., 2006; Sonda et al., 2010; Carranza et al., 2016). Thus, the cumulative evidence clearly implicates aspects of RNAi in *vsp* control, however the exact mechanism for how expression is limited to a single VSP remains unknown. For a more detailed review of the proposed theories, refer to (Gargantini et al., 2016).

### DNA Recombination and Repair

The DNA recombination and repair machinery are known to play an important role in the biology of multicopy gene families and mutually exclusive expression in multiple organisms, either by driving diversification of family members or through direct involvement in choosing which gene is expressed. In model systems, the two most studied examples are yeast mating-type switching and V(D)J recombination in the immunoglobulin loci. As previously mentioned, mating-type switching in yeast requires recombination of the locus where the active gene resides with one of two silent donor loci (Figure 1). In both yeast systems, the switch is initiated by a double strand break (DSB) at the active gene followed by Rad51/Rad52 mediated homologous recombination using one of the silent donor loci as the template for repair (Malone and Esposito, 1980; Aboussekhra et al., 1992). In *S. pombe* this DNA rearrangement results from two events during S-phase. The first, called imprint, involves the introduction of one or two ribonucleotides in a specific position (the MPS1 site) of the *mat1* locus during lagging-strand synthesis of DNA replication. This insertion is inherited by only one of the two daughter cells and during subsequent leading-strand synthesis, the imprint causes stalling of the replication fork, which triggers recombination between *mat1* and one of the two donor loci (*mat2P* or *mat3M*). As a result, one of the daughter cells has switched mating type (Dalgaard and Klar, 1999; Arcangioli and De Lahondes, 2000). In *S. cerevisiae*, the recombination event happens during G1 phase and is dependent on the expression of the endonuclease HO, which introduces a DSB at the *MAT* locus. As in *S. pombe*, this DSB is repaired by homologous recombination using the opposite mating-type donor cassette. The expression of HO is restricted to mother cells that have divided at least once (Strathern et al., 1982; Kostriken et al., 1983). For a recent detailed review of the mechanisms employed by both yeasts, *see* (Thon et al., 2019).

The tightly regulated action of the recombination machinery is also key for the generation of mature immunoglobulins. The process starts with the generation of DNA breaks at a specific sequence called the Recombination Signal Sequence (RSS) within one of the heavy chain alleles. This sequence lies next to each antigen receptor segment and it is crucial for recognition by the RAG1/2 recombinase complex (Swanson, 2004). Once one of the two alleles recombines successfully it produces a functional heavy chain that assembles with a surrogate light chain and forms the pre-B cell receptor. The pre-BCR initiates a feedback signal to inhibit rearrangement of the other heavy chain allele through the assembly of inaccessible chromatin and repression of RAG1/2. This also promotes a similar recombination at the light chain allele, including an analogous feedback to ensure only one allele is expressed and a mature B cell receptor is formed (detailed review in (Schatz and Ji, 2011)). Importantly, in order to have successful allelic exclusion, there must be asynchronicity between recombination of the two alleles, meaning that one of them has to recombine before the other. The choice of which allele recombines first is determined by differences in replication timing, with the allele that replicates early in S-phase undergoing the initial rearrangement (Mostoslavsky et al., 2001).

Regarding antigenic variation in parasites, recombination plays an especially prominent role for *vsg* gene expression in *T. brucei*. In addition to switching which expression site is activated, *T. brucei* has also evolved additional switching mechanisms involving DNA recombination (Figure 6). For example, the parasites can employ gene conversion to replace the entire active *vsg* gene with the *vsg* gene from a silent subtelomeric expression site. The homology region used for recombination can vary, leading to conversion events that can extend from the promoter region to the telomeric repeats. Alternatively, parasites can also undergo telomere exchange where the telomere ends, including the *vsg* expression sites, are exchanged without sequence loss. Lastly, multiple fragments from different *vsg* pseudogenes can be merged to form a “mosaic” *vsg* in a phenomenon referred to as segmental gene conversion (Myler et al., 1984; Hall et al., 2013; Mcculloch et al., 2015). Efficient recombination between intact *vsg* genes requires RAD51, BRCA2 and the homologous recombination machinery, although a minor amount of switching by conversion has been detected in the absence of RAD51 (Mcculloch and Barry, 1999; Hartley and Mcculloch, 2008). Similar to recombinational switching in yeast and immunoglobulin genes, it was demonstrated that switching by conversion is initiated by a DSB in the expression site (Boothroyd et al., 2009). While it is not clear what directs a DSB to occur specifically in the active expression site, it is possible that high transcriptional activity makes the locus more vulnerable to DNA lesions. It has been shown that RNA-DNA hybrids (R-loops) can accumulate at the active expression site, resulting in genomic instability (Briggs et al., 2019), and as described in the next section, the active ES was also shown to be depleted of nucleosomes. It was proposed that a combination of this depletion with high levels of transcription could result in natural DSBs (Figueiredo and Cross, 2010; Stanne and Rudenko, 2010).

The ability to recombine variant genes is not only important for switching which gene is expressed, but also to drive diversification of the family between different parasite strains. For example, *P. falciparum var* genes undergo much more rapid diversification than the rest of the genome, and this diversification results from frequent recombination between variant gene family members during asexual replication (Bopp et al., 2013; Claessens et al., 2014; Otto et al., 2018a). Since *Plasmodium* lacks components of nonhomologous end joining (NHEJ), it relies entirely on homologous recombination (HR) to repair DNA breaks (Kirkman et al., 2014). Additionally, since the parasites are haploid during their asexual stage, DSBs repaired by HR must use homologous sequences from elsewhere in the genome as template for repair. When such breaks occur at or near *var* genes, the creation of chimeric *var* genes through gene conversion events are favoured (Siao et al., 2020). Most *var* genes are also located in subtelomeric regions that are vulnerable to telomere healing and telomere exchange, which can result in cascades of recombination between *var* genes that ultimately give rise to new mosaic *var* genes and drive rapid diversification of the family (Zhang et al., 2019).

### Histones and Chromatin Modifications

A key component that all the described systems have in common is that activation and silencing of genes are associated with changes in chromatin modifications and assembly. Genes that need to be kept in a silent state are associated with silencing histone marks and heterochromatin formation, whereas expressed genes are associated with activating histone marks and a euchromatic state. Interestingly, for systems that employ recombination as the main means of gene switching, like yeast and immunoglobulin genes, the donor sequence is always associated with increased nuclease accessibility and activating histone modifications, such as acetylation (Klar et al., 1998; Bergman et al., 2003). The components involved in epigenetic control of multicopy gene families are largely conserved between the major model organisms. For example, gene activation is typically associated with acetylation on histone 3 and 4 (H3/H4ac), or with methylation of lysines 4 and 36 on histone 3 (H3K4me/HE3K36me). In contrast, gene silencing is usually driven by methylation on histone 3, including H3K9 or H3K27. Several conserved chromatin modifying enzymes are involved in this process. Among the most conserved are heterochromatin-associated protein HP1 (Swi6 in yeast), the histone demethylases Lsd1 and Lsd2, the nucleosome remodelling proteins SWI/SNF and the polycomb proteins PRC1/PRC2 (Ekwall et al., 1995; Okamoto et al., 2004; Holmes et al., 2012; Brockdorff, 2013; Jaeger et al., 2013; Tan et al., 2013; Ji et al., 2019).

As a consequence of its early divergence from the eukaryotic lineage, the chromatin organization and histone code of *Trypanosoma brucei* are only partially conserved. In addition to the canonical histones, *T. brucei* possesses four histone variants: H2Az, H2Bv, H3v and H4v. Additionally, the core histones appear to possess fewer modifications than in other eukaryotes, and the majority of them are not conserved at the sequence level (Maree and Patterton, 2014). Despite these divergences, epigenetic control has clearly been shown to be involved in activation and silencing of *vsg* expression. Silent *vsg* loci are enriched in H1, H2A, H3 and H3v, whereas the active expression site is depleted of nucleosomes (Figueiredo and Cross, 2010; Stanne and Rudenko, 2010). Additionally, a few chromatin modifiers have been implicated in *vsg* control. DOT1B is a histone methyltransferase that trimethylates lysine 76 of histone H3 in *T. brucei* and is required for *vsg* silencing (Figueiredo et al., 2008). Similarly, an orthologous member of the ISWI family of SWI2/SNF2-related chromatin-remodelling proteins (TbISWI) was demonstrated to be involved in downregulation of ES expression (Hughes et al., 2007), and knockdown of the chromatin remodeller and transcription elongation factor FACT/SPT16 results in permissive chromatin and *vsg* derepression (Denninger et al., 2010). *T. brucei* DNA is not methylated, but presents a modified base, β-D-glucopyranosyloxymethyluracil, called Base J (Gommers-Ampt et al., 1993). This base is enriched at trypanosome telomeres and at silent *vsg* genes but is not present at the active expression site (Van Leeuwen et al., 1997) and appears to be involved in termination of polycistronic transcription rather than transcriptional initiation (Reynolds et al., 2014). More recently, a protein complex specific to the active *vsg* was identified. VSG Exclusion 1 (VEX1) was shown to be sequestered at the active *vsg* locus and to associate with VEX2, an orthologue of the nonsense-mediated-decay helicase, UPF1. Together, this complex sustains chromatin accessibility and active transcription. Additionally, when the complex is depleted, *vsg* expression becomes heterogeneous (Glover et al., 2016; Faria et al., 2019).

In *P. falciparum*, the histone tails are largely conserved in sequence with those of model eukaryotes, however the linker histone H1 is missing (Gardner et al., 2002). Some additional histone variants have been identified, namely H2Az and H2Bz, with the latter seemingly unique to Apicomplexans. These two variant histones are associated with the activating histone marks H3K9ac and H3K4me3 and are enriched at the active *var* gene (Bartfai et al., 2010; Hoeijmakers et al., 2013; Petter et al., 2013). Additionally, *Plasmodium* has devoted specific histone marks, which are typically distributed throughout the genome in model organisms, to clonally variant gene families including *var* genes. Specifically, H3K9 trimethylation is enriched at the promoters of silent *var* genes and is recognized by the silencing heterochromatin protein 1 (PfHP1), whereas H3K36me3, deposited by the methyltransferase PfSet2, has been found on both silent and active *var* genes (Flueck et al., 2009; Salcedo-Amaya et al., 2009; Jiang et al., 2013; Ukaegbu et al., 2014). PfSet10, a methyltransferase responsible for methylation of H3K4, has been associated with *var* genes activation. Interestingly, this enzyme localizes to the active *var* gene during the late stage of asexual replication, when *var* genes are not expressed, so it has been proposed to play a role in maintenance of epigenetic memory through multiple cell cycles (Volz et al., 2012). ATAC-Seq has been used to analyse nucleosome distribution at *var* genes and detected several peaks of accessibility, but not a clear correlation with the active *var* gene. Interestingly, it was proposed that increased accessibility of the two *RUF-6* genes flanking the active *var* gene could play a role in *var* transcriptional activation (Ruiz et al., 2018).

In *G. lamblia*, the precise involvement of epigenetic control remains unclear. It appears that acetylation of the upstream region of *vsp* genes is associated with activation and it is known that RNA interference can induce the deposition of epigenetic marks (Kulakova et al., 2006; Francia, 2015). However, when histone deacetylases (HDACs) were knocked out, no major change was observed in *vsp* gene expression (Sonda et al., 2010).

### Nuclear Position and Chromatin Assembly

Spatial organization of DNA within the nucleus is crucial to gene regulation. In 1949 Barr and Bertram first reported a “nucleolar satellite” in the nucleus of feline nerve cells. This nuclear body was only found in cells isolated from female cats and was not observed in male cats. They went on to speculate that “the nucleolar satellite may be derived from the heterochromatin of the sex chromosomes.” (Barr and Bertram, 1949). This structure, known as the Barr body, was further determined to be formed by one of the two sex chromosomes in female cells (Ohno and Hauschka, 1960) leading to the hypothesis that this condensation resulted in silencing and inactivation of the chromosome (Lyon, 1961). The silenced X chromosome was shown to localize either to the nucleolus or the nuclear periphery (Barr and Bertram, 1949; Klinger and Schwarzacher, 1960), consistent with the heterochromatic positioning associated with transcriptional silencing (Towbin et al., 2013; Holla et al., 2020). Nuclear organization and assembly of silent chromatin have also been implicated in monoallelic expression of olfactory receptors in olfactory sensory neurons. Interchromosomal interactions and nuclear repositioning are key to the stabilization of the stochastic selection of a single OR from the thousands of available alleles (Chess et al., 1994). Following activation of a single OR gene, there is a confluence of the OR gene clusters (Armelin-Correa et al., 2014) resulting in increased interactions between the Greek Island enhancer elements and the active OR allele (Markenscoff-Papadimitriou et al., 2014; Monahan et al., 2019). This allows for the formation of a super-enhancer-like unique nuclear phase, allowing for the local accumulation of activating factors (Figure 4C). Key to the repression of the silent ORs is their aggregation into a small number of distinct heterochromatic foci (Clowney et al., 2012; Lyons et al., 2013).

Numerous lines of evidence also support the conclusion that correct recombination and assembly of the active immunoglobulin locus is guided by nuclear positioning (Reviewed in (Jhunjhunwala et al., 2009)). Consistent with what is observed for X chromosome inactivation and OR gene expression, in progenitor cells, the Ig loci are associated with the nuclear lamina and kept in a silent state. Subsequently in the gene undergoing recombination, chromatin looping allows the D and J segments to re-localize, while the V region remains tethered to the nuclear periphery. During B cell commitment, the V region is also relocated away from the nuclear lamina. Once productive rearrangement of the locus has occurred, the non-functional Ig allele repositioned into heterochromatin at the nuclear periphery (Skok et al., 2001; Kosak et al., 2002; Roldan et al., 2005).

Similar dynamics involving nuclear relocation and chromatin reorganization have been observed in parasites, including *T. brucei* which, as mentioned above, lacks identifiable enhancers that could mediate chromosome looping or repositioning. One of the first pieces of evidence in *T. brucei* was provided by the identification of an extra nucleolar RNA Polymerase I transcription site during the bloodstream stage of the parasite’s life cycle. This site, named the Expression Site body (ESB), is where the active ES is located and gets transcribed (Navarro and Gull, 2001). Remarkably, partial nuclear relocation is also observed for the silent ESs. During the bloodstream stage, the silent ESs are separated from the active ESB, but are located in extranucleolar clusters, whereas in the insect form all Es are sequestered in the nuclear heterochromatic periphery (Landeira and Navarro, 2007). The relocation of silent ESs during the bloodstream stage is proposed to promote more rapid activation. The ESB is maintained throughout the cell cycle and is dependent on the cohesin complex. Depletion of this complex interferes with antigenic switching and promotes activation of a previously silent ES (Landeira et al., 2009). Similarly, depletion of the nuclear periphery proteins 1 and 2 (NUP-1/2) results in increased VSG switching, providing additional evidence that nuclear positioning is crucial in trypanosome antigenic variation (Dubois et al., 2012; Maishman et al., 2016). In a recent publication, Budzak and others identified three different nuclear bodies associated with the ESB: the Cajal body, the spliced-leader array body (SLAB) and the NUF1P body. They proposed that these bodies facilitate the high requirement for splicing that needs to occur at this site (Budzak et al., 2022).

Nuclear localization is also thought to play an important role in control of *var* gene expression in *P. falciparum*. *var* genes are known to cluster at the nuclear periphery, although some argument remains as to whether all *var* genes cluster together in one locus, as suggested by recent Hi-C profiling, or instead in multiple spots as suggested by FISH experiments (Freitas-Junior et al., 2000; Freitas-Junior et al., 2005; Lemieux et al., 2013; Ay et al., 2014). Similar to *T. brucei*, the active *var* gene appears to relocalize into a discreet euchromatic location, separated from the silent *var* genes (Duraisingh et al., 2005; Ralph et al., 2005). The repositioning of the active *var* promoter to a different nuclear location was also demonstrated using extrachromosomal *var* promoters located on episomes (Dzikowski et al., 2007). These experiments confirmed that the active gene is separated from silent genes, but also showed that more than one promoter can localize within the active site at the same time, suggesting that localization alone is not enough to maintain mutually exclusive expression (Voss et al., 2006; Dzikowski and Deitsch, 2008). This conclusion was also supported by observations in parasites expressing two *var* genes at the same time (Joergensen et al., 2010). Interestingly, it was shown that active members of another clonally variant multicopy gene family, the *rifins*, relocate in the same active nuclear compartment as *var* genes (Howitt et al., 2009).

### A Two-step Process for Selecting a Gene for Activation

Models of mutually exclusive expression have typically presumed that activation of a single copy or allele is a strictly controlled process, where in every cell only one gene can be activated at any given time without exceptions. Recent technical advances, especially the ability to observe certain phenomena at single cell resolution, has partially challenged this dogma. Employing single cell RNA-sequencing, two independent groups analysed the transcriptomes of single olfactory sensory neurons during development in order to establish when OSNs select a single OR gene for expression. They discovered that before committing to expression of a single OR gene, immature OSNs express low levels of multiple genes. It is not known if the single gene that is ultimately fully activated is selected from the subset of genes that are initially expressed at a low level, a model described as “winner-take-all,” however, it is clear that selection of a single OR gene requires initial multiple gene transcription (Hanchate et al., 2015; Tan et al., 2015).

A similar phenomenon has recently been described in African trypanosomes prior to selection of a metacyclic *vsg* (m*vsg*) for expression. As described in the introduction, *T. brucei* begins to express a VSG coat when still in the insect host at the metacyclic stage (Tetley et al., 1987; Ramey-Butler et al., 2015). Hutchinson and others analysed single cell transcriptomes of parasites in the salivary glands of tsetse flies and identified the presence of two metacyclic populations: one, described as pre-metacyclic, expressing multiple mVSGs at low levels and one expressing a single mVSG at a higher level (Hutchinson et al., 2021). Additionally, in the mammalian host, single cell RNA-Seq and single cell RT-PCR showed that transcription initiation happens at several ES promoters in every cell, but that productive elongation only occurs at one (Kassem et al., 2014; Muller et al., 2018). In both systems, the choice of a single gene for activation is hypothesized to occur through a two-step process in which initially multiple genes are expressed followed by selection of a single gene in the fully mature cell. Both examples also highlight how studying certain phenomena at the population level is not sufficient to decipher all aspects of the underlying mechanisms in detail. For a recent review on the importance of cell-to-cell studies in *T. brucei*, check (Luzak et al., 2021).

Although analysis of *var* gene expression at the single cell level in *P. falciparum* has not yet been published, a two-step selection scenario similar to ORs and *vsg* has been hypothesized based on the study of clonal populations and mathematical modelling using data derived from *in vitro* cultures (Recker et al., 2011). This model describes an optimized hypothetical *var* gene network wherein parasites initially enter an intermediate “many” state in which several *var* genes are expressed at low levels. This is followed by selection and high-level activation of a single *var* gene, and the encoded PfEMP1 becomes the dominant antigen expressed by the population of parasites. In addition to these studies of cultured parasites, *in vivo* infections showed that at the onset of a bloodstream infection, multiple *var* genes are detectably transcribed at a low level (Wang et al., 2009; Bachmann et al., 2016). Similarly, it was demonstrated that erasing the epigenetic *var* memory by promoter titration or passage through the mosquito results in erythrocytic-stage parasites expressing a subset of *var* genes before establishment of mutually exclusive expression (Dzikowski et al., 2007; Fastman et al., 2012). These studies suggest that a two-step process for selection of a single gene for mutually exclusive expression might be a common pathway found throughout the eukaryotic lineage.

## Conclusion

Mutually exclusive expression of genes from multicopy families appears to be a strategy conserved throughout eukaryotic evolution. As described in this review, several layers of control interact in a complex mechanism that ultimately results in the expression of a single family member. Understanding the molecular details underlying mutually exclusive expression remains a challenge in all eukaryotes, from model systems to the evolutionarily distant protozoan parasites. Nonetheless the development of new methodologies and modern technological advances have shed new light on this puzzle and provided hints that many of the mechanisms involved are likely to be shared throughout the eukaryotic lineage. This represents an exciting time to work in this field given that more discoveries are likely as new methods are refined and are applied to additional biological systems.

With regard to the pathogenic organisms, while many basic strategies are shared it also is clear that the divergent nature of the parasites’ genomes has led to differences in some aspects of how antigenic variation is controlled. For example, the substantial difference in the size of the antigen encoding gene families in *T. brucei* when compared to *P. falciparum* could be partially responsible for the different strategies evolved by the two parasites. In addition, the polycistronic nature of kinetoplast transcription prevents *T. brucei* from relying solely on transcriptional regulation the way *P. falciparum* appears to. Similarly, the bi-nucleated structure of *G. lamblia* requires post-transcriptional control in the parasite’s cytoplasm, thus ensuring that only a single mRNA is expressed despite the existence of two transcriptionally active nuclei.

Despite these differences, much can be learned by exploring and comparing the strategies of different organisms. A better understanding of how mutually exclusive expression contributes to antigenic variation will undoubtedly improve our understanding of pathogenesis and virulence and might also translate into new disease intervention strategies. Thus, the advantages of comparative studies that apply the lessons learned in model systems to organisms of significance to human health continue to hold great promise.

## References

[B1] Aboussekhra A. Chanet R. Adjiri A. Fabre F. (1992). Semidominant Suppressors of Srs2 Helicase Mutations of *Saccharomyces cerevisiae* Map in the RAD51 Gene, Whose Sequence Predicts a Protein with Similarities to Procaryotic RecA Proteins. Mol. Cel Biol 12, 3224–3234. 10.1128/mcb.12.7.3224-3234.1992 PMC364537 1620127

[B2] Adam R. D. (2001). Biology of Giardia Lamblia. Clin. Microbiol. Rev. 14, 447–475. 10.1128/cmr.14.3.447-475.2001 11432808 PMC88984

[B3] Amit-Avraham I. Pozner G. Eshar S. Fastman Y. Kolevzon N. Yavin E. (2015). Antisense Long Noncoding RNAs Regulate Var Gene Activation in the Malaria Parasite Plasmodium Falciparum. Proc. Natl. Acad. Sci. USA 112, E982–E991. 10.1073/pnas.1420855112 25691743 PMC4352787

[B4] Arcangioli B. De Lahondes R. (2000). Fission Yeast Switches Mating Type by a Replication-Recombination Coupled Process. EMBO J. 19, 1389–1396. 10.1093/emboj/19.6.1389 10716938 PMC305679

[B5] Armelin-Correa L. M. Gutiyama L. M. Brandt D. Y. C. Malnic B. (2014). Nuclear Compartmentalization of Odorant Receptor Genes. Proc. Natl. Acad. Sci. 111, 2782–2787. 10.1073/pnas.1317036111 24550308 PMC3932893

[B6] Augui S. Nora E. P. Heard E. (2011). Regulation of X-Chromosome Inactivation by the X-Inactivation centre. Nat. Rev. Genet. 12, 429–442. 10.1038/nrg2987 21587299

[B7] Avner P. Heard E. (2001). X-chromosome Inactivation: Counting, Choice and Initiation. Nat. Rev. Genet. 2, 59–67. 10.1038/35047580 11253071

[B8] Avraham I. Schreier J. Dzikowski R. (2012). Insulator-like Pairing Elements Regulate Silencing and Mutually Exclusive Expression in the Malaria Parasite Plasmodium Falciparum. Proc. Natl. Acad. Sci. 109, E3678–E3686. 10.1073/pnas.1214572109 23197831 PMC3535642

[B9] Ay F. Bunnik E. M. Varoquaux N. Bol S. M. Prudhomme J. Vert J.-P. (2014). Three-dimensional Modeling of the P. Falciparum Genome during the Erythrocytic Cycle Reveals a strong Connection between Genome Architecture and Gene Expression. Genome Res. 24, 974–988. 10.1101/gr.169417.113 24671853 PMC4032861

[B10] Bachmann A. Petter M. Krumkamp R. Esen M. Held J. Scholz J. A. M. (2016). Mosquito Passage Dramatically Changes Var Gene Expression in Controlled Human Plasmodium Falciparum Infections. Plos Pathog. 12, e1005538. 10.1371/journal.ppat.1005538 27070311 PMC4829248

[B11] Barcons-Simon A. Cordon-Obras C. Guizetti J. Bryant J. M. Scherf A. (2020). CRISPR Interference of a Clonally Variant GC-Rich Noncoding RNA Family Leads to General Repression of Var Genes in Plasmodium Falciparum. *mBio* 11. 10.1128/mBio.03054-19 PMC6974570 31964736

[B12] Barr M. L. Bertram E. G. (1949). A Morphological Distinction between Neurones of the Male and Female, and the Behaviour of the Nucleolar Satellite during Accelerated Nucleoprotein Synthesis. Nature 163, 676–677. 10.1038/163676a0 18120749

[B13] Barry J. Graham S. V. Fotheringham M. Graham V. S. Kobryn K. Wymer B. (1998). VSG Gene Control and Infectivity Strategy of Metacyclic Stage Trypanosoma Brucei. Mol. Biochem. Parasitol. 91, 93–105. 10.1016/s0166-6851(97)00193-x 9574928

[B14] Bártfai R. Hoeijmakers W. A. M. Salcedo-Amaya A. M. Smits A. H. Janssen-Megens E. Kaan A. (2010). H2A.Z Demarcates Intergenic Regions of the Plasmodium Falciparum Epigenome that Are Dynamically Marked by H3K9ac and H3K4me3. Plos. Pathog. 6, e1001223. 10.1371/journal.ppat.1001223 21187892 PMC3002978

[B15] Bashkirova E. Lomvardas S. (2019). Olfactory Receptor Genes Make the Case for Inter-chromosomal Interactions. Curr. Opin. Genet. Development 55, 106–113. 10.1016/j.gde.2019.07.004 PMC6759391 31491591

[B16] Bergman Y. Fisher A. Cedar H. (2003). Epigenetic Mechanisms that Regulate Antigen Receptor Gene Expression. Curr. Opin. Immunol. 15, 176–181. 10.1016/s0952-7915(03)00016-5 12633667

[B17] Boddey J. A. Cowman A. F. (2013). Plasmodium Nesting: Remaking the Erythrocyte from the inside Out. Annu. Rev. Microbiol. 67, 243–269. 10.1146/annurev-micro-092412-155730 23808341

[B18] Boothroyd C. E. Dreesen O. Leonova T. Ly K. I. Figueiredo L. M. Cross G. A. M. (2009). A Yeast-Endonuclease-Generated DNA Break Induces Antigenic Switching in Trypanosoma Brucei. Nature 459, 278–281. 10.1038/nature07982 19369939 PMC2688456

[B19] Bopp S. E. R. Manary M. J. Bright A. T. Johnston G. L. Dharia N. V. Luna F. L. (2013). Mitotic Evolution of Plasmodium Falciparum Shows a Stable Core Genome but Recombination in Antigen Families. Plos. Genet. 9, e1003293. 10.1371/journal.pgen.1003293 23408914 PMC3567157

[B20] Briggs E. Crouch K. Lemgruber L. Hamilton G. Lapsley C. Mcculloch R. (2019). Trypanosoma Brucei Ribonuclease H2A Is an Essential R-Loop Processing Enzyme Whose Loss Causes DNA Damage during Transcription Initiation and Antigenic Variation. Nucleic Acids Res. 47, 9180–9197. 10.1093/nar/gkz644 31350892 PMC6753483

[B21] Broadbent K. M. Park D. Wolf A. R. Van Tyne D. Sims J. S. Ribacke U. (2011). A Global Transcriptional Analysis of Plasmodium Falciparum Malaria Reveals a Novel Family of Telomere-Associated lncRNAs. Genome Biol. 12, R56. 10.1186/gb-2011-12-6-r56 21689454 PMC3218844

[B22] Brockdorff N. (2013). Noncoding RNA and Polycomb Recruitment. RNA 19, 429–442. 10.1261/rna.037598.112 23431328 PMC3677253

[B23] Brugat T. Reid A. J. Lin J.-w. Cunningham D. Tumwine I. Kushinga G. (2017). Antibody-independent Mechanisms Regulate the Establishment of Chronic Plasmodium Infection. Nat. Microbiol. 2, 16276. 10.1038/nmicrobiol.2016.276 28165471 PMC5373435

[B24] Buck L. Axel R. (1991). A Novel Multigene Family May Encode Odorant Receptors: a Molecular Basis for Odor Recognition. Cell 65, 175–187. 10.1016/0092-8674(91)90418-x 1840504

[B25] Budzak J. Jones R. Tschudi C. Kolev N. G. Rudenko G. (2022). An Assembly of Nuclear Bodies Associates with the Active VSG Expression Site in African Trypanosomes. Nat. Commun. 13, 101. 10.1038/s41467-021-27625-6 35013170 PMC8748868

[B26] Calderwood M. S. Gannoun-Zaki L. Wellems T. E. Deitsch K. W. (2003). Plasmodium Falciparum Var Genes Are Regulated by Two Regions with Separate Promoters, One Upstream of the Coding Region and a Second within the Intron. J. Biol. Chem. 278, 34125–34132. 10.1074/jbc.m213065200 12832422

[B27] Carranza P. G. Gargantini P. R. Prucca C. G. Torri A. Saura A. Svärd S. (2016). Specific Histone Modifications Play Critical Roles in the Control of Encystation and Antigenic Variation in the Early-Branching Eukaryote Giardia Lamblia. Int. J. Biochem. Cel Biol. 81, 32–43. 10.1016/j.biocel.2016.10.010 27771437

[B28] Chess A. Simon I. Cedar H. Axel R. (1994). Allelic Inactivation Regulates Olfactory Receptor Gene Expression. Cell 78, 823–834. 10.1016/s0092-8674(94)90562-2 8087849

[B29] Claessens A. Hamilton W. L. Kekre M. Otto T. D. Faizullabhoy A. Rayner J. C. (2014). Generation of Antigenic Diversity in Plasmodium Falciparum by Structured Rearrangement of Var Genes during Mitosis. Plos. Genet. 10, e1004812. 10.1371/journal.pgen.1004812 25521112 PMC4270465

[B30] Clayton C. (2019). Regulation of Gene Expression in Trypanosomatids: Living with Polycistronic Transcription. Open Biol. 9, 190072. 10.1098/rsob.190072 31164043 PMC6597758

[B31] Clowney E. J. Legros M. A. Mosley C. P. Clowney F. G. Markenskoff-Papadimitriou E. C. Myllys M. (2012). Nuclear Aggregation of Olfactory Receptor Genes Governs Their Monogenic Expression. Cell 151, 724–737. 10.1016/j.cell.2012.09.043 23141535 PMC3659163

[B32] Corcoran A. E. (2010). The Epigenetic Role of Non-coding RNA Transcription and Nuclear Organization in Immunoglobulin Repertoire Generation. Semin. Immunol. 22, 353–361. 10.1016/j.smim.2010.08.001 20863715

[B33] Cortes A. Deitsch K. W. (2017). Malaria Epigenetics. Cold Spring Harb. Perspect. Med. 10.1101/cshperspect.a025528 PMC5495052 28320828

[B34] Cowman A. F. Healer J. Marapana D. Marsh K. (2016). Malaria: Biology and Disease. Cell 167, 610–624. 10.1016/j.cell.2016.07.055 27768886

[B35] Cross G. A. M. (1975). Identification, Purification and Properties of Clone-specific Glycoprotein Antigens Constituting the Surface Coat ofTrypanosoma Brucei. Parasitology 71, 393–417. 10.1017/s003118200004717x 645

[B36] Dalgaard J. Z. Klar A. J. S. (1999). Orientation of DNA Replication Establishes Mating-type Switching Pattern in *S. pombe* . Nature 400, 181–184. 10.1038/22139 10408447

[B37] Dalgaard J. Z. Vengrova S. (2004). Selective Gene Expression in Multigene Families from Yeast to Mammals. Sci. STKE 2004, re17. 10.1126/stke.2562004re17 15507595

[B38] Deitsch K. W. Calderwood M. S. Wellems T. E. (2001). Cooperative Silencing Elements in Var Genes. Nature 412, 875–876. 10.1038/35091146 11528468

[B39] Deitsch K. W. Dzikowski R. (2017). Variant Gene Expression and Antigenic Variation by Malaria Parasites. Annu. Rev. Microbiol. 71, 625–641. 10.1146/annurev-micro-090816-093841 28697665

[B40] Denninger V. Fullbrook A. Bessat M. Ersfeld K. Rudenko G. (2010). The FACT Subunit TbSpt16 Is Involved in Cell Cycle Specific Control of VSG Expression Sites in Trypanosoma Brucei. Mol. Microbiol. 78, 459–474. 10.1111/j.1365-2958.2010.07350.x 20879999 PMC3034197

[B41] Dubois K. N. Alsford S. Holden J. M. Buisson J. Swiderski M. Bart J.-M. (2012). NUP-1 Is a Large Coiled-Coil Nucleoskeletal Protein in Trypanosomes with Lamin-like Functions. Plos Biol. 10, e1001287. 10.1371/journal.pbio.1001287 22479148 PMC3313915

[B42] Duraisingh M. T. Horn D. (2016). Epigenetic Regulation of Virulence Gene Expression in Parasitic Protozoa. Cell Host & Microbe 19, 629–640. 10.1016/j.chom.2016.04.020 27173931 PMC5061559

[B43] Duraisingh M. T. Voss T. S. Marty A. J. Duffy M. F. Good R. T. Thompson J. K. (2005). Heterochromatin Silencing and Locus Repositioning Linked to Regulation of Virulence Genes in Plasmodium Falciparum. Cell 121, 13–24. 10.1016/j.cell.2005.01.036 15820675

[B44] Dzikowski R. Deitsch K. W. (2008). Active Transcription Is Required for Maintenance of Epigenetic Memory in the Malaria Parasite Plasmodium Falciparum. J. Mol. Biol. 382, 288–297. 10.1016/j.jmb.2008.07.015 18656490 PMC3614407

[B45] Dzikowski R. Li F. Amulic B. Eisberg A. Frank M. Patel S. (2007). Mechanisms Underlying Mutually Exclusive Expression of Virulence Genes by Malaria Parasites. EMBO Rep. 8, 959–965. 10.1038/sj.embor.7401063 17762879 PMC2002552

[B46] Early P. Huang H. Davis M. Calame K. Hood L. (1980). An Immunoglobulin Heavy Chain Variable Region Gene Is Generated from Three Segments of DNA: VH, D and JH. Cell 19, 981–992. 10.1016/0092-8674(80)90089-6 6769593

[B47] Ekwall K. Javerzat J.-P. Lorentz A. Schmidt H. Cranston G. Allshire R. (1995). The Chromodomain Protein Swi6: a Key Component at Fission Yeast Centromeres. Science 269, 1429–1431. 10.1126/science.7660126 7660126

[B48] Engreitz J. M. Pandya-Jones A. Mcdonel P. Shishkin A. Sirokman K. Surka C. (2013). The Xist lncRNA Exploits Three-Dimensional Genome Architecture to Spread across the X Chromosome. Science 341, 1237973. 10.1126/science.1237973 23828888 PMC3778663

[B49] Epp C. Li F. Howitt C. A. Chookajorn T. Deitsch K. W. (2009). Chromatin Associated Sense and Antisense Noncoding RNAs Are Transcribed from the Var Gene Family of Virulence Genes of the Malaria Parasite Plasmodium Falciparum. RNA 15, 116–127. 10.1261/rna.1080109 19037012 PMC2612763

[B50] Faria J. Glover L. Hutchinson S. Boehm C. Field M. C. Horn D. (2019). Monoallelic Expression and Epigenetic Inheritance Sustained by a Trypanosoma Brucei Variant Surface Glycoprotein Exclusion Complex. Nat. Commun. 10, 3023. 10.1038/s41467-019-10823-8 31289266 PMC6617441

[B51] Faria J. Luzak V. Müller L. S. M. Brink B. G. Hutchinson S. Glover L. (2021). Spatial Integration of Transcription and Splicing in a Dedicated Compartment Sustains Monogenic Antigen Expression in African Trypanosomes. Nat. Microbiol. 6, 289–300. 10.1038/s41564-020-00833-4 33432154 PMC7610597

[B52] Fastman Y. Noble R. Recker M. Dzikowski R. (2012). Erasing the Epigenetic Memory and Beginning to Switch-The Onset of Antigenic Switching of Var Genes in Plasmodium Falciparum. Plos. ONE 7, e34168. 10.1371/journal.pone.0034168 22461905 PMC3312910

[B53] Figueiredo L. M. Cross G. A. M. (2010). Nucleosomes Are Depleted at the VSG Expression Site Transcribed by RNA Polymerase I in African Trypanosomes. Eukaryot. Cel 9, 148–154. 10.1128/ec.00282-09 PMC2805297 19915072

[B54] Figueiredo L. M. Freitas-Junior L. H. Bottius E. Olivo-Marin J. C. Scherf A. (2002). A central Role for Plasmodiumfalciparum Subtelomeric Regions in Spatial Positioning and Telomere Length Regulation. Embo J. 21, 815–824. 10.1093/emboj/21.4.815 11847128 PMC125872

[B55] Figueiredo L. M. Janzen C. J. Cross G. A. M. (2008). A Histone Methyltransferase Modulates Antigenic Variation in African Trypanosomes. Plos Biol. 6, e161. 10.1371/journal.pbio.0060161 18597556 PMC2443197

[B56] Flueck C. Bartfai R. Volz J. Niederwieser I. Salcedo-Amaya A. M. Alako B. T. F. (2009). Plasmodium Falciparum Heterochromatin Protein 1 marks Genomic Loci Linked to Phenotypic Variation of Exported Virulence Factors. Plos. Pathog. 5, e1000569. 10.1371/journal.ppat.1000569 19730695 PMC2731224

[B57] Francia S. (2015). Non-Coding RNA: Sequence-specific Guide for Chromatin Modification and DNA Damage Signaling. Front. Genet. 6, 320. 10.3389/fgene.2015.00320 26617633 PMC4643122

[B58] Frank M. Dzikowski R. Costantini D. Amulic B. Berdougo E. Deitsch K. (2006). Strict Pairing of Var Promoters and Introns Is Required for Var Gene Silencing in the Malaria Parasite Plasmodium Falciparum. J. Biol. Chem. 281, 9942–9952. 10.1074/jbc.m513067200 16455655 PMC3941977

[B59] Freitas-Junior L. H. Bottius E. Pirrit L. A. Deitsch K. W. Scheidig C. Guinet F. (2000). Frequent Ectopic Recombination of Virulence Factor Genes in Telomeric Chromosome Clusters of P. Falciparum. Nature 407, 1018–1022. 10.1038/35039531 11069183

[B60] Freitas-Junior L. H. Hernandez-Rivas R. Ralph S. A. Montiel-Condado D. Ruvalcaba-Salazar O. K. Rojas-Meza A. P. (2005). Telomeric Heterochromatin Propagation and Histone Acetylation Control Mutually Exclusive Expression of Antigenic Variation Genes in Malaria Parasites. Cell 121, 25–36. 10.1016/j.cell.2005.01.037 15820676

[B61] Froberg J. E. Yang L. Lee J. T. (2013). Guided by RNAs: X-Inactivation as a Model for lncRNA Function. J. Mol. Biol. 425, 3698–3706. 10.1016/j.jmb.2013.06.031 23816838 PMC3771680

[B62] Fuss S. H. Omura M. Mombaerts P. (2007). Local and Cis Effects of the H Element on Expression of Odorant Receptor Genes in Mouse. Cell 130, 373–384. 10.1016/j.cell.2007.06.023 17662950

[B63] Gardner M. J. Hall N. Fung E. White O. Berriman M. Hyman R. W. (2002). Genome Sequence of the Human Malaria Parasite Plasmodium Falciparum. Nature 419, 498–511. 10.1038/nature01097 12368864 PMC3836256

[B64] Gargantini P. R. Serradell M. d. C. Ríos D. N. Tenaglia A. H. Luján H. D. (2016). Antigenic Variation in the Intestinal Parasite Giardia Lamblia. Curr. Opin. Microbiol. 32, 52–58. 10.1016/j.mib.2016.04.017 27177351

[B65] Ginger M. L. Blundell P. A. Lewis A. M. Browitt A. Günzl A. Barry J. D. (2002). Ex Vivo and In Vitro Identification of a Consensus Promoter for VSG Genes Expressed by Metacyclic-Stage Trypanosomes in the Tsetse Fly. Eukaryot. Cel 1, 1000–1009. 10.1128/ec.1.6.1000-1009.2002 PMC138762 12477800

[B66] Glover L. Hutchinson S. Alsford S. Horn D. (2016). VEX1 Controls the Allelic Exclusion Required for Antigenic Variation in Trypanosomes. Proc. Natl. Acad. Sci. USA 113, 7225–7230. 10.1073/pnas.1600344113 27226299 PMC4932947

[B67] Glover L. Hutchinson S. Alsford S. Mcculloch R. Field M. C. Horn D. (2013). Antigenic Variation in A Frican Trypanosomes: the Importance of Chromosomal and Nuclear Context in VSG Expression Control. Cell Microbiol 15, 1984–1993. 10.1111/cmi.12215 24047558 PMC3963442

[B68] Glusman G. Yanai I. Rubin I. Lancet D. (2001). The Complete Human Olfactory Subgenome. Genome Res. 11, 685–702. 10.1101/gr.171001 11337468

[B69] Goldmit M. Bergman Y. (2004). Monoallelic Gene Expression: a Repertoire of Recurrent Themes. Immunol. Rev. 200, 197–214. 10.1111/j.0105-2896.2004.00158.x 15242406

[B70] Gommers-Ampt J. H. Van Leeuwen F. De Beer A. L. J. Vliegenthart J. F. G. Dizdaroglu M. Kowalak J. A. (1993). β-d-glucosyl-hydroxymethyluracil: A Novel Modified Base Present in the DNA of the Parasitic Protozoan T. Brucei: a Novel Modified Base Present in the DNA of the Parasitic Protozoan. Cell 75, 1129–1136. 10.1016/0092-8674(93)90322-h 8261512

[B71] Graves J. A. M. (2006). Sex Chromosome Specialization and Degeneration in Mammals. Cell 124, 901–914. 10.1016/j.cell.2006.02.024 16530039

[B72] Graves J. A. M. (1995). The Origin and Function of the Mammalian Y Chromosome and Y-Borne Genes - an Evolving Understanding. Bioessays 17, 311–320. 10.1002/bies.950170407 7741724

[B73] Gross M. R. Hsu R. Deitsch K. W. (2021). Evolution of Transcriptional Control of Antigenic Variation and Virulence in Human and Ape Malaria Parasites. BMC Ecol. Evo 21, 139. 10.1186/s12862-021-01872-z PMC8265125 34238209

[B74] Guizetti J. Barcons-Simon A. Scherf A. (2016). Trans-acting GC-Rich Non-coding RNA at Var Expression Site Modulates Gene Counting in Malaria Parasite. Nucleic Acids Res. 44, 9710–9718. 10.1093/nar/gkw664 27466391 PMC5175341

[B75] Günzl A. Bruderer T. Laufer G. Schimanski B. Tu L. C. Chung H. M. (2003). RNA Polymerase I Transcribes Procyclin Genes and Variant Surface Glycoprotein Gene Expression Sites in Trypanosoma Brucei. Eukaryot. Cel 2, 542–551. 10.1128/ec.2.3.542-551.2003 PMC161450 12796299

[B76] Haber J. E. (1998). Mating-type Gene Switching in *Saccharomyces cerevisiae* . Annu. Rev. Genet. 32, 561–599. 10.1146/annurev.genet.32.1.561 9928492

[B77] Hall J. P. J. Wang H. Barry J. D. (2013). Mosaic VSGs and the Scale of Trypanosoma Brucei Antigenic Variation. Plos Pathog. 9, e1003502. 10.1371/journal.ppat.1003502 23853603 PMC3708902

[B78] Hanchate N. K. Kondoh K. Lu Z. Kuang D. Ye X. Qiu X. (2015). Single-cell Transcriptomics Reveals Receptor Transformations during Olfactory Neurogenesis. Science 350, 1251–1255. 10.1126/science.aad2456 26541607 PMC5642900

[B79] Hartley C. L. Mcculloch R. (2008). Trypanosoma Brucei BRCA2 Acts in Antigenic Variation and Has Undergone a Recent Expansion in BRC Repeat Number that Is Important during Homologous Recombination. Mol. Microbiol. 68, 1237–1251. 10.1111/j.1365-2958.2008.06230.x 18430140 PMC2408642

[B80] Hoeijmakers W. A. M. Salcedo‐Amaya A. M. Smits A. H. Françoijs K. J. Treeck M. Gilberger T. W. (2013). H 2 A . Z / H 2 B . Z Double‐variant Nucleosomes Inhabit the at ‐rich Promoter Regions of the P Lasmodium Falciparum Genome. Mol. Microbiol. 87, 1061–1073. 10.1111/mmi.12151 23320541 PMC3594968

[B81] Holla S. Dhakshnamoorthy J. Folco H. D. Balachandran V. Xiao H. Sun L.-l. (2020). Positioning Heterochromatin at the Nuclear Periphery Suppresses Histone Turnover to Promote Epigenetic Inheritance. Cell 180, 150–164. 10.1016/j.cell.2019.12.004 31883795 PMC7102895

[B82] Holmes A. Roseaulin L. Schurra C. Waxin H. Lambert S. Zaratiegui M. (2012). Lsd1 and Lsd2 Control Programmed Replication fork Pauses and Imprinting in Fission Yeast. Cel Rep. 2, 1513–1520. 10.1016/j.celrep.2012.10.011 PMC3909218 23260662

[B83] Horn D. (2014). Antigenic Variation in African Trypanosomes. Mol. Biochem. Parasitol. 10.1016/j.molbiopara.2014.05.001 PMC4155160 24859277

[B84] Howitt C. A. Wilinski D. Llinás M. Templeton T. J. Dzikowski R. Deitsch K. W. (2009). Clonally Variant Gene Families inPlasmodium Falciparumshare a Common Activation Factor. Mol. Microbiol. 73, 1171–1185. 10.1111/j.1365-2958.2009.06846.x 19708920 PMC2752644

[B85] Hughes K. Wand M. Foulston L. Young R. Harley K. Terry S. (2007). A Novel ISWI Is Involved in VSG Expression Site Downregulation in African Trypanosomes. EMBO J. 26, 2400–2410. 10.1038/sj.emboj.7601678 17431399 PMC1864976

[B86] Hutchinson S. Foulon S. Crouzols A. Menafra R. Rotureau B. Griffiths A. D. (2021). The Establishment of Variant Surface Glycoprotein Monoallelic Expression Revealed by Single-Cell RNA-Seq of Trypanosoma Brucei in the Tsetse Fly Salivary Glands. Plos Pathog. 17, e1009904. 10.1371/journal.ppat.1009904 34543350 PMC8509897

[B87] Jaeger S. Fernandez B. Ferrier P. (2013). Epigenetic Aspects of Lymphocyte Antigen Receptor Gene Rearrangement or 'when Stochasticity Completes Randomness'. Immunology 139, 141–150. 10.1111/imm.12057 23278765 PMC3647179

[B88] Jakočiūnas T. Holm L. R. Verhein-Hansen J. Trusina A. Thon G. (2013). Two Portable Recombination Enhancers Direct Donor Choice in Fission Yeast Heterochromatin. Plos Genet. 9, e1003762. 10.1371/journal.pgen.1003762 24204285 PMC3812072

[B89] Jhunjhunwala S. Van Zelm M. C. Peak M. M. Murre C. (2009). Chromatin Architecture and the Generation of Antigen Receptor Diversity. Cell 138, 435–448. 10.1016/j.cell.2009.07.016 19665968 PMC2726833

[B90] Ji Z. Sheng Y. Miao J. Li X. Zhao H. Wang J. (2019). The Histone Methyltransferase Setd2 Is Indispensable for V(D)J Recombination. Nat. Commun. 10, 3353. 10.1038/s41467-019-11282-x 31350389 PMC6659703

[B91] Jia S. Yamada T. Grewal S. I. S. (2004). Heterochromatin Regulates Cell Type-specific Long-Range Chromatin Interactions Essential for Directed Recombination. Cell 119, 469–480. 10.1016/j.cell.2004.10.020 15537537

[B92] Jiang L. Mu J. Zhang Q. Ni T. Srinivasan P. Rayavara K. (2013). PfSETvs Methylation of Histone H3K36 Represses Virulence Genes in Plasmodium Falciparum. Nature 499, 223–227. 10.1038/nature12361 23823717 PMC3770130

[B93] Joergensen L. Bengtsson D. C. Bengtsson A. Ronander E. Berger S. S. Turner L. (2010). Surface Co-expression of Two Different PfEMP1 Antigens on Single Plasmodium Falciparum-Infected Erythrocytes Facilitates Binding to ICAM1 and PECAM1. Plos. Pathog. 6, e1001083. 10.1371/journal.ppat.1001083 20824088 PMC2932717

[B94] Jung D. Alt F. W. (2004). Unraveling V(D)J Recombination. Cell 116, 299–311. 10.1016/s0092-8674(04)00039-x 14744439

[B95] Kassem A. Pays E. Vanhamme L. (2014). Transcription Is Initiated on Silent Variant Surface Glycoprotein Expression Sites Despite Monoallelic Expression in Trypanosoma Brucei. Proc. Natl. Acad. Sci. 111, 8943–8948. 10.1073/pnas.1404873111 24889641 PMC4066500

[B96] Kelly M. Burke J. Smith M. Klar A. Beach D. (1988). Four Mating-type Genes Control Sexual Differentiation in the Fission Yeast. EMBO J. 7, 1537–1547. 10.1002/j.1460-2075.1988.tb02973.x 2900761 PMC458406

[B97] Kirkman L. A. Lawrence E. A. Deitsch K. W. (2014). Malaria Parasites Utilize Both Homologous Recombination and Alternative End Joining Pathways to Maintain Genome Integrity. Nucleic Acids Res. 42, 370–379. 10.1093/nar/gkt881 24089143 PMC3874194

[B98] Klar A. J. Ivanova A. V. Dalgaard J. Z. Bonaduce M. J. Grewal S. I. (1998). Multiple Epigenetic Events Regulate Mating-type Switching of Fission Yeast. Novartis Found. Symp. 214, 87–103. 10.1002/9780470515501.ch6 9601013

[B99] Klar A. J. S. Hicks J. B. Strathern J. N. (1982). Directionality of Yeast Mating-type Interconversion. Cell 28, 551–561. 10.1016/0092-8674(82)90210-0 7042099

[B100] Klar A. J. S. (1990). Regulation of Fission Yeast Mating-type Interconversion by Chromosome Imprinting. Dev. Suppl. 108, 3–8. 10.1242/dev.108.supplement.3 2090428

[B101] Klinger H. P. Schwarzacher H. G. (1960). The Sex Chromatin and Heterochromatic Bodies in Human Diploid and Polyploid Nuclei. J. Biophys. Biochem. Cytol. 8, 345–364. 10.1083/jcb.8.2.345 13756884 PMC2224938

[B102] Kooter J. Van Der Spek H. J. Wagter R. D'oliveira C. E. Van Der Hoeven F. Johnson P. J. (1987). The Anatomy and Transcription of a Telomeric Expression Site for Variant-specific Surface Antigens in T. Brucei. Cell 51, 261–272. 10.1016/0092-8674(87)90153-x 2444341

[B103] Kosak S. T. Skok J. A. Medina K. L. Riblet R. Le Beau M. M. Fisher A. G. (2002). Subnuclear Compartmentalization of Immunoglobulin Loci during Lymphocyte Development. Science 296, 158–162. 10.1126/science.1068768 11935030

[B104] Kostriken R. Strathern J. N. Klar A. J. S. Hicks J. B. Heffron F. (1983). A Site-specific Endonuclease Essential for Mating-type Switching in *Saccharomyces cerevisiae* . Cell 35, 167–174. 10.1016/0092-8674(83)90219-2 6313222

[B105] Kulakova L. Singer S. M. Conrad J. Nash T. E. (2006). Epigenetic Mechanisms Are Involved in the Control of Giardia Lamblia Antigenic Variation. Mol. Microbiol. 61, 1533–1542. 10.1111/j.1365-2958.2006.05345.x 16968226

[B106] Kyes S. A. Christodoulou Z. Raza A. Horrocks P. Pinches R. Rowe J. A. (2003). A Well-Conserved Plasmodium Falciparum Var Gene Shows an Unusual Stage-specific Transcript Pattern. Mol. Microbiol. 48, 1339–1348. 10.1046/j.1365-2958.2003.03505.x 12787360 PMC2869446

[B107] Lahn B. T. Page D. C. (1997). Functional Coherence of the Human Y Chromosome. Science 278, 675–680. 10.1126/science.278.5338.675 9381176

[B108] Landeira D. Bart J.-M. Van Tyne D. Navarro M. (2009). Cohesin Regulates VSG Monoallelic Expression in Trypanosomes. J. Cel Biol 186, 243–254. 10.1083/jcb.200902119 PMC2717648 19635842

[B109] Landeira D. Navarro M. (2007). Nuclear Repositioning of the VSG Promoter during Developmental Silencing in Trypanosoma Brucei. J. Cel Biol 176, 133–139. 10.1083/jcb.200607174 PMC2063932 17210949

[B110] Lee J. Davidow L. S. Warshawsky D. (1999). Tsix, a Gene Antisense to Xist at the X-Inactivation centre. Nat. Genet. 21, 400–404. 10.1038/7734 10192391

[B111] Lee J. T. Strauss W. M. Dausman J. A. Jaenisch R. (1996). A 450 Kb Transgene Displays Properties of the Mammalian X-Inactivation center. Cell 86, 83–94. 10.1016/s0092-8674(00)80079-3 8689690

[B112] Lemieux J. E. Kyes S. A. Otto T. D. Feller A. I. Eastman R. T. Pinches R. A. (2013). Genome‐wide Profiling of Chromosome Interactions in P Lasmodium Falciparum Characterizes Nuclear Architecture and Reconfigurations Associated with Antigenic Variation. Mol. Microbiol. 90, 519–537. 10.1111/mmi.12381 23980881 PMC3894959

[B113] Lennon G. G. Perry R. P. (1985). Cμ-containing Transcripts Initiate Heterogeneously within the IgH Enhancer Region and Contain a Novel 5′-nontranslatable Exon. Nature 318, 475–478. 10.1038/318475a0 3934561

[B114] Li B. (2015). DNA Double-Strand Breaks and Telomeres Play Important Roles in Trypanosoma Brucei Antigenic Variation. Eukaryot. Cel 14, 196–205. 10.1128/ec.00207-14 PMC4346566 25576484

[B115] Li X. Fu X.-D. (2019). Chromatin-associated RNAs as Facilitators of Functional Genomic Interactions. Nat. Rev. Genet. 20, 503–519. 10.1038/s41576-019-0135-1 31160792 PMC7684979

[B116] Lin J.-w. Reid A. J. Cunningham D. Böhme U. Tumwine I. Keller-Mclaughlin S. (2018). Genomic and Transcriptomic Comparisons of Closely Related Malaria Parasites Differing in Virulence and Sequestration Pattern. Wellcome Open Res. 3, 142. 10.12688/wellcomeopenres.14797.1 30542666 PMC6259598

[B117] Loda A. Heard E. (2019). Xist RNA in Action: Past, Present, and Future. Plos Genet. 15, e1008333. 10.1371/journal.pgen.1008333 31537017 PMC6752956

[B118] Lomvardas S. Barnea G. Pisapia D. J. Mendelsohn M. Kirkland J. Axel R. (2006). Interchromosomal Interactions and Olfactory Receptor Choice. Cell 126, 403–413. 10.1016/j.cell.2006.06.035 16873069

[B119] Lukes J. Skalicky T. Tyc J. Votypka J. Yurchenko V. (2014). Evolution of Parasitism in Kinetoplastid Flagellates. Mol. Biochem. Parasitol. 195, 115–122. 24893339 10.1016/j.molbiopara.2014.05.007

[B120] Luzak V. López-Escobar L. Siegel T. N. Figueiredo L. M. (2021). Cell-to-Cell Heterogeneity in Trypanosomes. Annu. Rev. Microbiol. 75, 107–128. 10.1146/annurev-micro-040821-012953 34228491

[B121] Lyon M. F. (1961). Gene Action in the X-Chromosome of the Mouse (*Mus musculus* L.). Nature 190, 372–373. 10.1038/190372a0 13764598

[B122] Lyons D. B. Allen W. E. Goh T. Tsai L. Barnea G. Lomvardas S. (2013). An Epigenetic Trap Stabilizes Singular Olfactory Receptor Expression. Cell 154, 325–336. 10.1016/j.cell.2013.06.039 23870122 PMC3929589

[B123] Maclary E. Hinten M. Harris C. Kalantry S. (2013). Long Nonoding RNAs in the X-Inactivation center. Chromosome Res. 21, 601–614. 10.1007/s10577-013-9396-2 24297756 PMC3919162

[B124] Maishman L. Obado S. O. Alsford S. Bart J.-M. Chen W.-M. Ratushny A. V. (2016). Co-dependence between Trypanosome Nuclear Lamina Components in Nuclear Stability and Control of Gene Expression. Nucleic Acids Res. 44, 10554–10570. 10.1093/nar/gkw751 27625397 PMC5159534

[B125] Malnic B. Hirono J. Sato T. Buck L. B. (1999). Combinatorial Receptor Codes for Odors. Cell 96, 713–723. 10.1016/s0092-8674(00)80581-4 10089886

[B126] Malone R. E. Esposito R. E. (1980). The RAD52 Gene Is Required for Homothallic Interconversion of Mating Types and Spontaneous Mitotic Recombination in Yeast. Proc. Natl. Acad. Sci. 77, 503–507. 10.1073/pnas.77.1.503 6987653 PMC348300

[B127] Maree J. P. Patterton H.-G. (2014). The Epigenome of Trypanosoma Brucei: a Regulatory Interface to an Unconventional Transcriptional Machine. Biochim. Biophys. Acta (Bba) - Gene Regul. Mech. 1839, 743–750. 10.1016/j.bbagrm.2014.05.028 PMC4138444 24942804

[B128] Markenscoff-Papadimitriou E. Allen W. E. Colquitt B. M. Goh T. Murphy K. K. Monahan K. (2014). Enhancer Interaction Networks as a Means for Singular Olfactory Receptor Expression. Cell 159, 543–557. 10.1016/j.cell.2014.09.033 25417106 PMC4243057

[B129] Maxfield Boumil R. Lee J. T. (2001). Forty Years of Decoding the Silence in X-Chromosome Inactivation. Hum. Mol. Genet. 10, 2225–2232. 10.1093/hmg/10.20.2225 11673405

[B130] Mcculloch R. Morrison L. J. Hall J. P. J. (2015). DNA Recombination Strategies during Antigenic Variation in the African Trypanosome. Microbiol. Spectr. 3, MDNA3. 10.1128/microbiolspec.MDNA3-0016-2014 26104717

[B131] Mcculloch R. Barry J. D. (1999). A Role for RAD51 and Homologous Recombination in Trypanosoma Brucei Antigenic Variation. Genes Development 13, 2875–2888. 10.1101/gad.13.21.2875 10557214 PMC317127

[B132] Mok B. W. Ribacke U. Rasti N. Kironde F. Chen Q. Nilsson P. (2008). Default Pathway of Var2csa Switching and Translational Repression in Plasmodium Falciparum. Plos. ONE 3, e1982. 10.1371/journal.pone.0001982 18431472 PMC2292259

[B133] Monahan K. Horta A. Lomvardas S. (2019). LHX2- and LDB1-Mediated Trans Interactions Regulate Olfactory Receptor Choice. Nature 565, 448–453. 10.1038/s41586-018-0845-0 30626972 PMC6436840

[B134] Monahan K. Lomvardas S. (2015). Monoallelic Expression of Olfactory Receptors. Annu. Rev. Cel Dev. Biol. 31, 721–740. 10.1146/annurev-cellbio-100814-125308 PMC4882762 26359778

[B135] Morrison H. G. Mcarthur A. G. Gillin F. D. Aley S. B. Adam R. D. Olsen G. J. (2007). Genomic Minimalism in the Early Diverging Intestinal Parasite Giardia Lamblia. Science 317, 1921–1926. 10.1126/science.1143837 17901334

[B136] Mostoslavsky R. Singh N. Tenzen T. Goldmit M. Gabay C. Elizur S. (2001). Asynchronous Replication and Allelic Exclusion in the Immune System. Nature 414, 221–225. 10.1038/35102606 11700561

[B137] Müller L. S. M. Cosentino R. O. Förstner K. U. Guizetti J. Wedel C. Kaplan N. (2018). Genome Organization and DNA Accessibility Control Antigenic Variation in Trypanosomes. Nature 563, 121–125. 10.1038/s41586-018-0619-8 30333624 PMC6784898

[B138] Myler P. J. Allison J. Agabian N. Stuart K. (1984). Antigenic Variation in African Trypanosomes by Gene Replacement or Activation of Alternate Telomeres. Cell 39, 203–211. 10.1016/0092-8674(84)90206-x 6091912

[B139] Nash T. E. Aggarwal A. (1986). Cytotoxicity of Monoclonal Antibodies to a Subset of Giardia Isolates. J. Immunol. 136, 2628–2632. 3950421

[B140] Nash T. E. (2002). Surface Antigenic Variation in Giardia Lamblia. Mol. Microbiol. 45, 585–590. 10.1046/j.1365-2958.2002.03029.x 12139606

[B141] Navarro M. Gull K. (2001). A Pol I Transcriptional Body Associated with VSG Mono-Allelic Expression in Trypanosoma Brucei. Nature 414, 759–763. 10.1038/414759a 11742402

[B142] Nelson R. G. Parsons M. Barr P. J. Stuart K. Selkirk M. Agabian N. (1983). Sequences Homologous to the Variant Antigen mRNA Spliced Leader Are Located in Tandem Repeats and Variable Orphons in Trypanosoma Brucei. Cell 34, 901–909. 10.1016/0092-8674(83)90547-0 6313213

[B143] Niimura Y. Nei M. (2003). Evolution of Olfactory Receptor Genes in the Human Genome. Proc. Natl. Acad. Sci. 100, 12235–12240. 10.1073/pnas.1635157100 14507991 PMC218742

[B144] Niimura Y. Nei M. (2007). Extensive Gains and Losses of Olfactory Receptor Genes in Mammalian Evolution. PLoS One 2, e708. 10.1371/journal.pone.0000708 17684554 PMC1933591

[B145] Ogawa Y. Lee J. T. (2003). Xite, X-Inactivation Intergenic Transcription Elements that Regulate the Probability of Choice. Mol. Cel 11, 731–743. 10.1016/s1097-2765(03)00063-7 12667455

[B146] Ohno S. Hauschka T. S. (1960). Allocycly of the X-Chromosome in Tumors and normal Tissues. Cancer Res. 20, 541–545. 14428472

[B147] Okamoto I. Otte A. P. Allis C. D. Reinberg D. Heard E. (2004). Epigenetic Dynamics of Imprinted X Inactivation during Early Mouse Development. Science 303, 644–649. 10.1126/science.1092727 14671313

[B148] Otto T. D. Böhme U. Jackson A. P. Hunt M. Franke-Fayard B. Hoeijmakers W. A. M. (2014). A Comprehensive Evaluation of Rodent Malaria Parasite Genomes and Gene Expression. BMC. Biol. 12, 86. 10.1186/s12915-014-0086-0 25359557 PMC4242472

[B149] Otto T. D. Böhme U. Sanders M. J. Reid A. J. Bruske E. I. Duffy C. W. (2018a). Long Read Assemblies of Geographically Dispersed Plasmodium Falciparum Isolates Reveal Highly Structured Subtelomeres. Wellcome. Open. Res. 3, 52. 10.12688/wellcomeopenres.14571.1 29862326 PMC5964635

[B150] Otto T. D. Gilabert A. Crellen T. Böhme U. Arnathau C. Sanders M. (2018b). Genomes of All Known Members of a Plasmodium Subgenus Reveal Paths to Virulent Human Malaria. Nat. Microbiol. 3, 687–697. 10.1038/s41564-018-0162-2 29784978 PMC5985962

[B151] Penny G. D. Kay G. F. Sheardown S. A. Rastan S. Brockdorff N. (1996). Requirement for Xist in X Chromosome Inactivation. Nature 379, 131–137. 10.1038/379131a0 8538762

[B152] Perry K. L. Watkins K. P. Agabian N. (1987). Trypanosome mRNAs Have Unusual "cap 4" Structures Acquired by Addition of a Spliced Leader. Proc. Natl. Acad. Sci. 84, 8190–8194. 10.1073/pnas.84.23.8190 3120186 PMC299507

[B153] Petter M. Selvarajah S. A. Lee C. C. Chin W. H. Gupta A. P. Bozdech Z. (2013). H2A.Z and H2B.Z Double-Variant Nucleosomes Define Intergenic Regions and Dynamically Occupyvargene Promoters in the Malaria parasitePlasmodium Falciparum. Mol. Microbiol. 87, 1167–1182. 10.1111/mmi.12154 23373537

[B154] Plath K. Mlynarczyk-Evans S. Nusinow D. A. Panning B. (2002). Xist RNA and the Mechanism of X Chromosome Inactivation. Annu. Rev. Genet. 36, 233–278. 10.1146/annurev.genet.36.042902.092433 12429693

[B155] Pourmorady A. Lomvardas S. (2021). Olfactory Receptor Choice: a Case Study for Gene Regulation in a Multi-Enhancer System. Curr. Opin. Genet. Dev. 72, 101–109. 10.1016/j.gde.2021.11.003 34896807 PMC12734978

[B156] Prucca C. G. Lujan H. D. (2009). Antigenic Variation inGiardia Lamblia. Cel Microbiol 11, 1706–1715. 10.1111/j.1462-5822.2009.01367.x 19709056

[B157] Prucca C. G. Slavin I. Quiroga R. Elías E. V. Rivero F. D. Saura A. (2008). Antigenic Variation in Giardia Lamblia Is Regulated by RNA Interference. Nature 456, 750–754. 10.1038/nature07585 19079052

[B158] Ralph S. A. Scheidig-Benatar C. Scherf A. (2005). Antigenic Variation in Plasmodium Falciparum Is Associated with Movement of Var Loci between Subnuclear Locations. Proc. Natl. Acad. Sci. 102, 5414–5419. 10.1073/pnas.0408883102 15797990 PMC556247

[B159] Ramey-Butler K. Ullu E. Kolev N. G. Tschudi C. (2015). Synchronous Expression of Individual Metacyclic Variant Surface Glycoprotein Genes in Trypanosoma Brucei. Mol. Biochem. Parasitol. 200, 1–4. 10.1016/j.molbiopara.2015.04.001 25896436 PMC4470760

[B160] Recker M. Buckee C. O. Serazin A. Kyes S. Pinches R. Christodoulou Z. (2011). Antigenic Variation in Plasmodium Falciparum Malaria Involves a Highly Structured Switching Pattern. Plos. Pathog. 7, e1001306. 10.1371/journal.ppat.1001306 21408201 PMC3048365

[B161] Reynolds D. Cliffe L. Förstner K. U. Hon C.-C. Siegel T. N. Sabatini R. (2014). Regulation of Transcription Termination by Glucosylated Hydroxymethyluracil, Base J, in Leishmania Major and Trypanosoma Brucei. Nucleic Acids Res. 42, 9717–9729. 10.1093/nar/gku714 25104019 PMC4150806

[B162] Roldán E. Fuxa M. Chong W. Martinez D. Novatchkova M. Busslinger M. (2005). Locus 'decontraction' and Centromeric Recruitment Contribute to Allelic Exclusion of the Immunoglobulin Heavy-Chain Gene. Nat. Immunol. 6, 31–41. 10.1038/ni1150 15580273 PMC1592471

[B163] Rudenko G. Blundell P. A. Dirks-Mulder A. Kieft R. Borst P. (1995). A Ribosomal DNA Promoter Replacing the Promoter of a Telomeric VSG Gene Expression Site Can Be Efficiently Switched on and off in T. Brucei. Cell 83, 547–553. 10.1016/0092-8674(95)90094-2 7585957

[B164] Ruiz J. L. Tena J. J. Bancells C. Cortés A. Gómez-Skarmeta J. L. Gómez-Díaz E. (2018). Characterization of the Accessible Genome in the Human Malaria Parasite Plasmodium Falciparum. Nucleic Acids Res. 46, 9414–9431. 10.1093/nar/gky643 30016465 PMC6182165

[B165] Salcedo-Amaya A. M. Van Driel M. A. Alako B. T. Trelle M. B. Van Den Elzen A. M. G. Cohen A. M. (2009). Dynamic Histone H3 Epigenome Marking during the Intraerythrocytic Cycle of Plasmodium Falciparum. Proc. Natl. Acad. Sci. 106, 9655–9660. 10.1073/pnas.0902515106 19497874 PMC2701018

[B166] Saraiya A. A. Li W. Wu J. Chang C. H. Wang C. C. (2014). The microRNAs in an Ancient Protist Repress the Variant-specific Surface Protein Expression by Targeting the Entire Coding Sequence. Plos. Pathog. 10, e1003791. 10.1371/journal.ppat.1003791 24586143 PMC3937270

[B167] Schatz D. G. Ji Y. (2011). Recombination Centres and the Orchestration of V(D)J Recombination. Nat. Rev. Immunol. 11, 251–263. 10.1038/nri2941 21394103

[B168] Scherf A. Hernandez-Rivas R. Buffet P. Bottius E. Benatar C. Pouvelle B. (1998). Antigenic Variation in Malaria: *In Situ* Switching, Relaxed and Mutually Exclusive Transcription of *Var* Genes during Intra-erythrocytic Development in Plasmodium Falciparum. EMBO J. 17, 5418–5426. 10.1093/emboj/17.18.5418 9736619 PMC1170867

[B169] Serizawa S. Miyamichi K. Nakatani H. Suzuki M. Saito M. Yoshihara Y. (2003). Negative Feedback Regulation Ensures the One Receptor-One Olfactory Neuron Rule in Mouse. Science 302, 2088–2094. 10.1126/science.1089122 14593185

[B170] Siao M. C. B. Perkins S. L. Deitsch K. W. Kirkman L. A. (2020). Evolution of Host Specificity by Malaria Parasites through Altered Mechanisms Controlling Genome Maintenance. mBio 11, e03272–03219. 10.1128/mbio.03272-19 32184256 PMC7078485

[B171] Sierra-Miranda M. Delgadillo D. M. Mancio-Silva L. Vargas M. Villegas-Sepulveda N. Martínez-Calvillo S. (2012). Two Long Non-coding RNAs Generated from Subtelomeric Regions Accumulate in a Novel Perinuclear Compartment in Plasmodium Falciparum. Mol. Biochem. Parasitol. 185, 36–47. 10.1016/j.molbiopara.2012.06.005 22721695 PMC7116675

[B172] Skok J. A. Brown K. E. Azuara V. Caparros M.-L. Baxter J. Takacs K. (2001). Nonequivalent Nuclear Location of Immunoglobulin Alleles in B Lymphocytes. Nat. Immunol. 2, 848–854. 10.1038/ni0901-848 11526401

[B173] Sonda S. Morf L. Bottova I. Baetschmann H. Rehrauer H. Caflisch A. (2010). Epigenetic Mechanisms Regulate Stage Differentiation in the Minimized protozoanGiardia Lamblia. Mol. Microbiol. 76, 48–67. 10.1111/j.1365-2958.2010.07062.x 20132448

[B174] Stanne T. M. Rudenko G. (2010). Active VSG Expression Sites in Trypanosoma Brucei Are Depleted of Nucleosomes. Eukaryot. Cel 9, 136–147. 10.1128/ec.00281-09 PMC2805301 19915073

[B175] Strathern J. N. Klar A. J. S. Hicks J. B. Abraham J. A. Ivy J. M. Nasmyth K. A. (1982). Homothallic Switching of Yeast Mating Type Cassettes Is Initiated by a Double-Stranded Cut in the MAT Locus. Cell 31, 183–192. 10.1016/0092-8674(82)90418-4 6297747

[B176] Swanson P. C. (2004). The Bounty of RAGs: Recombination Signal Complexes and Reaction Outcomes. Immunol. Rev. 200, 90–114. 10.1111/j.0105-2896.2004.00159.x 15242399

[B177] Tan L. Li Q. Xie X. S. (2015). Olfactory Sensory Neurons Transiently Express Multiple Olfactory Receptors during Development. Mol. Syst. Biol. 11, 844. 10.15252/msb.20156639 26646940 PMC4704490

[B178] Tan L. Zong C. Xie X. S. (2013). Rare Event of Histone Demethylation Can Initiate Singular Gene Expression of Olfactory Receptors. Proc. Natl. Acad. Sci. 110, 21148–21152. 10.1073/pnas.1321511111 24344257 PMC3876194

[B179] Thon G. Maki T. Haber J. E. Iwasaki H. (2019). Mating-type Switching by Homology-Directed Recombinational Repair: a Matter of Choice. Curr. Genet. 65, 351–362. 10.1007/s00294-018-0900-2 30382337 PMC6420890

[B180] Towbin B. D. Gonzalez-Sandoval A. Gasser S. M. (2013). Mechanisms of Heterochromatin Subnuclear Localization. Trends Biochem. Sci. 38, 356–363. 10.1016/j.tibs.2013.04.004 23746617

[B193] Tetley L. Turner C. M. Barry J. D. Crowe J. S. Vickerman K. (1987). Onset of Expression of the Variant Surface Glycoproteins of *Trypanosoma brucei* in the tsetse fly studied using immunoelectron microscopy. J. Cell Sci. 87, 363–72. 10.1242/jcs.87.2.363 3654788

[B181] Ukaegbu U. E. Kishore S. P. Kwiatkowski D. L. Pandarinath C. Dahan-Pasternak N. Dzikowski R. (2014). Recruitment of PfSET2 by RNA Polymerase II to Variant Antigen Encoding Loci Contributes to Antigenic Variation in P. Falciparum. Plos. Pathog. 10, e1003854. 10.1371/journal.ppat.1003854 24391504 PMC3879369

[B182] Ukaegbu U. E. Zhang X. Heinberg A. R. Wele M. Chen Q. Deitsch K. W. (2015). A Unique Virulence Gene Occupies a Principal Position in Immune Evasion by the Malaria Parasite Plasmodium Falciparum. Plos. Genet. 11, e1005234. 10.1371/journal.pgen.1005234 25993442 PMC4437904

[B183] Upadhyay R. Bawankar P. Malhotra D. Patankar S. (2005). A Screen for Conserved Sequences with Biased Base Composition Identifies Noncoding RNAs in the A-T Rich Genome of Plasmodium Falciparum. Mol. Biochem. Parasitol. 144, 149–158. 10.1016/j.molbiopara.2005.08.012 16183147

[B184] Van Leeuwen F. Wijsman E. R. Kieft R. Van Der Marel G. A. Van Boom J. H. Borst P. (1997). Localization of the Modified Base J in Telomeric VSG Gene Expression Sites ofTrypanosoma Brucei. Genes Dev. 11, 3232–3241. 10.1101/gad.11.23.3232 9389654 PMC316749

[B185] Volz J. C. Bártfai R. Petter M. Langer C. Josling G. A. Tsuboi T. (2012). PfSET10, a Plasmodium Falciparum Methyltransferase, Maintains the Active Var Gene in a Poised State during Parasite Division. Cell Host & Microbe 11, 7–18. 10.1016/j.chom.2011.11.011 22264509

[B186] Voss T. S. Healer J. Marty A. J. Duffy M. F. Thompson J. K. Beeson J. G. (2006). A Var Gene Promoter Controls Allelic Exclusion of Virulence Genes in Plasmodium Falciparum Malaria. Nature 439, 1004–1008. 10.1038/nature04407 16382237

[B187] Wang C. W. Hermsen C. C. Sauerwein R. W. Arnot D. E. Theander T. G. Lavstsen T. (2009). The Plasmodium Falciparum Var Gene Transcription Strategy at the Onset of Blood Stage Infection in a Human Volunteer. Parasitol. Int. 58, 478–480. 10.1016/j.parint.2009.07.004 19616120

[B188] Wei G. Zhao Y. Zhang Q. Pan W. (2015). Dual Regulatory Effects of Non-coding GC-Rich Elements on the Expression of Virulence Genes in Malaria Parasites. Infect. Genet. Evol. 36, 490–499. 10.1016/j.meegid.2015.08.023 26299885

[B189] Weigert M. Gatmaitan L. Loh E. Schilling J. Hood L. (1978). Rearrangement of Genetic Information May Produce Immunoglobulin Diversity. Nature 276, 785–790. 10.1038/276785a0 103003

[B190] Wu X. Haber J. E. (1996). A 700 Bp Cis-Acting Region Controls Mating-type Dependent Recombination along the Entire Left Arm of Yeast Chromosome III. Cell 87, 277–285. 10.1016/s0092-8674(00)81345-8 8861911

[B191] Wutz A. Jaenisch R. (2000). A Shift from Reversible to Irreversible X Inactivation Is Triggered during ES Cell Differentiation. Mol. Cel 5, 695–705. 10.1016/s1097-2765(00)80248-8 10882105

[B192] Zhang X. Alexander N. Leonardi I. Mason C. Kirkman L. A. Deitsch K. W. (2019). Rapid Antigen Diversification through Mitotic Recombination in the Human Malaria Parasite Plasmodium Falciparum. Plos. Biol. 17, e3000271. 10.1371/journal.pbio.3000271 31083650 PMC6532940

